# T-type calcium channels cause bursts of spikes in motor but not sensory thalamic neurons during mimicry of natural patterns of synaptic input

**DOI:** 10.3389/fncel.2015.00428

**Published:** 2015-11-04

**Authors:** Haram R. Kim, Su Z. Hong, Christopher D. Fiorillo

**Affiliations:** Department of Bio and Brain Engineering, KAISTDaejeon, South Korea

**Keywords:** thalamocortical, natural statistics, pattern generation, predictive homeostasis, predictive coding, prediction error, temporal decorrelation, lateral geniculate

## Abstract

Although neurons within intact nervous systems can be classified as ‘sensory’ or ‘motor,’ it is not known whether there is any general distinction between sensory and motor neurons at the cellular or molecular levels. Here, we extend and test a theory according to which activation of certain subtypes of voltage-gated ion channel (VGC) generate patterns of spikes in neurons of motor systems, whereas VGC are proposed to counteract patterns in sensory neurons. We previously reported experimental evidence for the theory from visual thalamus, where we found that T-type calcium channels (TtCCs) did not cause bursts of spikes but instead served the function of ‘predictive homeostasis’ to maximize the causal and informational link between retinogeniculate excitation and spike output. Here, we have recorded neurons in brain slices from eight sensory and motor regions of rat thalamus while mimicking key features of natural excitatory and inhibitory post-synaptic potentials. As predicted by theory, TtCC did cause bursts of spikes in motor thalamus. TtCC-mediated responses in motor thalamus were activated at more hyperpolarized potentials and caused larger depolarizations with more spikes than in visual and auditory thalamus. Somatosensory thalamus is known to be more closely connected to motor regions relative to auditory and visual thalamus, and likewise the strength of its TtCC responses was intermediate between these regions and motor thalamus. We also observed lower input resistance, as well as limited evidence of stronger hyperpolarization-induced (‘H-type’) depolarization, in nuclei closer to motor output. These findings support our theory of a specific difference between sensory and motor neurons at the cellular level.

## Introduction

The distinction between ‘sensory’ and ‘motor’ is fundamental to popular conceptions of the nervous system. Neurons are conventionally categorized as ‘sensory’ or ‘motor’ based either on the relative number of feedforward synapses that separate them from sensory and motor peripheries, or the degree to which their firing is correlated with sensory vs. motor events. The value of this classification could be purely descriptive, and even misleading insofar as the semantic implication of a binary classification is at odds with the graded classification along a continuum that is applied in practice based on anatomy or physiology. More fundamentally, the sensory-motor distinction has no known basis at the cellular or molecular levels. It is conceivable that network connectivity within the intact nervous system is the sole determinant of the extent to which a neuron is better described as ‘sensory’ or ‘motor.’

The popularity of the sensory-motor distinction may have arisen from the intuitive notion that sensory perception is a passive process caused by the external world, whereas muscle contraction is caused by active, internally generated “commands” that are only partially dependent on current sensory events. Indeed, motor output is substantially caused by processes internal to the nervous system that are at least partially independent of current sensory input, extreme examples being “central pattern generators” (Marder and Bucher, 2007) and the fact that firing in motoneurons drives muscle contraction in the complete absence of sensory input early in development (Sanes et al., 2011). In principle, motor output should be caused by a combination of current sensory input and internally stored “prior” information (about which actions have value given the current sensory context). By contrast, accurate sensory perception should rely on a strict causal link between current external sensory events and spikes in sensory neurons.

We proposed that this sensory-motor dichotomy is present at the level of single neurons (Fiorillo, 2013; Fiorillo et al., 2014). According to theory, excitatory post-synaptic potentials (EPSPs) conveying current sensory evidence should ideally be the sole proximate cause of spikes in sensory neurons (Sherman and Guillery, 1998; Kay and Phillips, 2011; Fiorillo et al., 2014). We previously tested this by examining the function of T-type calcium channel (TtCC) in thalamocortical (TC) neurons of the lateral geniculate nucleus (LGN) while mimicking natural conditions. We found that TtCC amplified EPSPs toward spike threshold so that EPSPs may or may not cause spikes, depending on their amplitude (Hong et al., 2014). This supports our theory, according to which TtCC in sensory neurons should help to maximize the causal link between synaptic excitation and spikes without themselves causing spikes, through a process we have termed “predictive homeostasis” (Fiorillo et al., 2014; Hong et al., 2014). In this model of a sensory neuron, TtCC are one of many voltage-gated ion channel (VGC) subtypes predicting and counteracting sensory-related synaptic drive so that each spike signals a positive prediction error, in the spirit of “predictive coding” (e.g., Barlow, 1961). Here, we test the hypothesis that, in contrast to sensory TC neurons, TtCC in motor TC neurons will sum with synaptic excitation to directly cause spikes.

Testing this hypothesis is more challenging than it may initially appear. The greatest challenge is the fact that there is remarkable variability in intrinsic membrane properties across neurons that is clearly not related to any sensory-motor distinction. Although this variability is critical to our general framework for understanding neurons (Fiorillo, 2008; Fiorillo et al., 2014), it is unwanted “noise” with respect to the present hypothesis. We chose thalamus because TC neurons across functionally diverse regions are sufficiently similar to be considered a “single type” of neuron (Jahnsen and Llinás, 1984a,b). This helped to reduce variability in membrane properties related to other factors so that we could better isolate differences along the sensory-motor dimension.

A second major challenge is that the hypothesis concerns naturally occurring patterns of synaptic conductances that are experienced by neurons *in vivo*. *In vitro* recordings are typically performed under conditions of artificially high membrane excitability, particularly due to an absence of synaptic conductances (Destexhe et al., 2003). TtCC cause bursts of spikes in sensory TC neurons when synaptic conductances are absent (Jahnsen and Llinás, 1984a,b; Zhan et al., 1999), but cause few if any bursts when they are present (Wolfart et al., 2005; Deleuze et al., 2012; Hong et al., 2014). We have used dynamic clamp to mimic natural synaptic conductances.

## Materials and Methods

Frequently used abbreviations are listed in **Table 1**, including recorded nuclei.

**Table 1:** Abbreviations.

Recorded thalamic nuclei	
LGN	Lateral geniculate nucleus, dorsal division
LP	Lateral posterior
MD	Mediodorsal
MGv	Medial geniculate, ventral division
MGd	Medial geniculate, dorsal division
PoM	Posterior medial
VB	Ventrobasal
VL	Ventrolateral
**Others**	

aEPSG	Artificial excitatory post-synaptic conductance
CCSW	Current clamp square-wave protocol
DCSW	Dynamic clamp square-wave protocol
LTS	Low-threshold spike
fLTS	First LTS (the LTS caused by minimal current)
TC	Thalamocortical
TtCC	T-type calcium channel
TtD	T-type depolarization
VGC	Voltage-gated channel

### Animals

Brains were taken from male Sprague-Dawley rats euthanized with CO_2_ as part of a procedure approved by the KAIST Institutional Animal Care and Use Committee. Rats ranged in age from 21 to 35 days, but 379 of 450 neurons (84%) were recorded in rats of more than 28 days. Our intention was to compare TtCC-mediated responses across mature thalamic nuclei. Development of thalamus appears to be substantially complete by postnatal day 21, including TtCC-mediated responses and other intrinsic membrane properties (Ramoa and McCormick, 1994; Warren and Jones, 1997).

### Brain Slice Preparation and Whole-cell Patch Recordings

All methods of slice preparation and recording were described previously, including the dynamic clamp technique (Hong et al., 2014). During recordings, coronal slices (250 μm) were submerged in artificial cerebrospinal fluid (34°) containing (mM) 125 NaCl_2_, 2.5 KCl, 1.25 NaH_2_PO_4_, 25 NaHCO_3_, 1 MgCl_2_, 2 CaCl_2_, 10 D-glucose. Borosilicate pipettes (3–4 MΩ, 280 milli-osmoles) contained 135 K-methylsulfate, 1.5 MgCl_2_, 0.5 EGTA, 10 HEPES, 2 Mg-ATP, 0.2 Na_2_-GTP, and 10 phosphocreatine, with pH adjusted to 7.3 by KOH. Resting membrane potentials were recorded within ∼1 min after achieving the whole-cell configuration. Input resistance was measured by injecting hyperpolarizing currents of -20 pA for 150 ms from resting potential.

### Artificial Conductances

Details of the artificial conductances used in DCSW and aEPSG protocols were provided previously (Hong et al., 2014). Only key points are summarized here, starting with the naturally occurring patterns that we sought to mimic.

Under natural conditions, TtCC are activated at times when membrane conductance is high due to synaptic conductances. In LGN, GABA-mediated synaptic conductance causes hyperpolarization and deinactivates TtCC, and glutamate-mediated synaptic conductance can then cause depolarization and TtCC activation (Wang et al., 2007). The synaptically driven hyperpolarization is transient, with an average duration of about 250 ms or less (Wang et al., 2007). Most if not all TC neurons receive powerful synaptic excitation from just one or a small number of “driver” synapses (Sherman and Guillery, 1998; Sherman, 2007, 2012), and single action potentials from a single presynaptic axon are known to cause large unitary EPSPs in LGN (Turner and Salt, 1998; Blitz and Regehr, 2005; Sincich et al., 2007; Wang et al., 2007; Weyand, 2007). Under natural conditions *in vivo* the amplitude of retinogeniculate EPSPs shows remarkably little variation (Sincich et al., 2007; Wang et al., 2007; Weyand, 2007; Casti et al., 2008; Borst, 2010), in contrast to the powerful paired-pulse depression observed *in vitro* (see Discussion in Borst, 2010). Temporal summation of retinogeniculate EPSPs within a window of ∼30 ms is the primary variable that determines spike generation. Thus an EPSP occurring more than 30 ms after the previous EPSP almost never causes a spike, but a spike is almost always generated when the interval is less than 10 ms (Sincich et al., 2007; Weyand, 2007).

In DCSW experiments, a 10 nS conductance was used to mimic the summed conductance of both the synaptic inhibition that causes hyperpolarization as well as the synaptic excitation that activates TtCC (since depolarizing currents were not accompanied by any additional conductance). In aEPSG experiments, the artificial inhibitory conductance (3–10 nS, mean: 6 nS) was adjusted in each cell to be equal to the cell’s resting conductance and thus reduce total membrane resistance by half (excluding the contribution of EPSG). Previous efforts to mimic *in vivo* conditions by application of artificial conductances also reduced membrane resistance by about one half (Wolfart et al., 2005; Deleuze et al., 2012), or more (Ulrich and Huguenard, 1997a). To insure that membrane voltage was near -80 mV, the reversal potential of the artificial conductance was adjusted between -85 and -100 mV in each cell. We adjusted aEPSG amplitude so that the second but not the first of two aEPSG of equivalent unitary amplitude (separated by 5 ms) would cause one spike from a potential of -65 mV. This resulted in a range of unitary aEPSG peak amplitudes across cells of 7–12 nS. Our aEPSG waveform was designed to mimic only the AMPA-receptor-mediated component of retinogeniculate EPSG, and not the more complex NMDA-receptor component.

### Depolarizing Square-wave Currents

Currents were of 10 ms duration and covered a total range of 10–1000 pA. A more limited range was tested in some cells (**Table 2**). Current increments were 50 pA in the CCSW protocol (except 10 pA over a limited range in some cells; **Table 2**), which caused a voltage increment of 2.5 ± 0.7 mV (mean ± SD across VL and LGN, measured at the end of the 10 ms current injected from a baseline voltage of -70 mV). This voltage increment limited the resolution of all our measures of the fLTS, including its voltage threshold. The smaller the voltage increment, the greater our chance to observe a LTS that was graded with respect to amplitude or spike generation. We therefore chose a smaller increment of 10 pA in DCSW experiments, which caused a voltage increment of 0.3 ± 0.1 mV in response to currents injected from -65 mV.

**Table 2:** Protocols and numbers of recorded neurons.

Protocol/Base (mV)	Time (s)	Input range + Increment (pA)	VL	LGN	VB	MD	MGv	MGd	LP	PoM
			**131**	**177**	**57**	**33**	**21**	**10**	**9**	**12**
**CCSW**			*68*	*69*	*10*	*20*	*10*	*10*	*9*	*12*
-70, -80, -90	3.0	50 – 700, +50	31	20	10	20	10	10	9	12
-70, -80, -90	3.0	10 – 700, +10, +50	13	32						
-70, -80	3.0	150 – 700, +10, +50	24	17						
**DCSW**			*29*	*43*	*24*	*12*	*11*			
-80	0.2, 0.4, 0.8	200 - 500 , +10			13	12	11			
-75, -80	0.05, 0.2, 0.4, 0.8	200 – 500 (700), +10	9(3)	11(4)						
-80	0.05, 0.2, 0.4, 0.8	200 – 500 (700), +10	9(3)	2(5)	11					
-65, -80	0.05, 0.1, 0.2, 0.4, 0.8	100 – 500 , +10		4						
-65, -80	0.05, 0.1, 0.2, 0.4, 0.8	100 – 1000 , +10	11	26						
**aEPSG**			*19*	*51*	*16*					
-65, -80	0.05, 0.2, 0.4, 0.8	1, 2, and 4 aEPSG	15	13	16					
-65, -80	0.05, 0.1, 0.2, 0.4, 0.8	1, 2, and 4 aEPSG	4	38						
**Other**			*15*	*14*	*7*	*1*				

*Protocols were revised over time. We chose not to discard data, resulting in a complex data set. Bold numbers indicate total neurons, whereas italic numbers indicate totals within a protocol. “Time” refers to the intervals between depolarizing currents (CCSW), or to delays from onset of the hyperpolarizing conductance (DCSW and aEPSG). Parentheses indicate the number of additional neurons tested up to a maximum current of 700 pA. “Other” neurons were not recorded in any of the three protocols and contributed data only to passive membrane properties.*

### Temporal Aspects of Experimental Designs

In the CCSW protocol, membrane voltage was kept near -70, -80, or -90 mV for ∼1 min, except for one depolarizing current pulse (10 ms duration) delivered every 3.0 s. A base voltage near -80 mV was tested first, followed by -70 and then -90 mV. DCSW and aEPSG protocols consisted of alternating periods of a depolarized state near -65 mV (2.0 s) and a hyperpolarized state near -80 mV (1.0–1.2 s). In the DCSW protocol, one current amplitude was tested during each 3.0 s sweep, proceeding from smallest to largest over successive sweeps. The full range of current increments was tested after 50 ms near -80 mV in each cell before proceeding to test currents at 800, 100, 400, and 200 ms. In the aEPSG protocol, each of the three event types (1, 2, and 4 EPSG) was tested at each of the five delays following hyperpolarization in 15 consecutive ‘sweeps’ within a single ‘block,’ starting with one and progressing to four EPSG. Within each block, delays were tested in the order 50, 800, 100, 400, and 200 ms. The block was then repeated four times, for a total of four repetitions of each condition (EPSG count and delay) at -80 mV. For further details of the design, see Hong et al. (2014).

### Identification of Thalamocortical Neurons

Thalamocortical neurons are the majority in all regions of thalamus, and they can be readily distinguished from inter neurons by established criteria (McCormick and Pape, 1988; Williams et al., 1996). Prior to patching, a neuron was selected that had a large soma with more than two proximal dendrites. TC neurons were subsequently distinguished from interneurons by the presence of a rebound LTS with a burst of multiple sodium spikes in response to sudden offset of a large hyperpolarizaing current (indicative of T-type current) and a depolarizing ‘sag’ following strong hyperpolarization (indicative of H-type current). Neurons in VL and LGN were occasionally observed that lacked these properties and had very high input resistance (as much as 1000 MΩ). Data was not collected from these neurons.

### Anatomical Localization of Neurons

**FIGURE 1:**
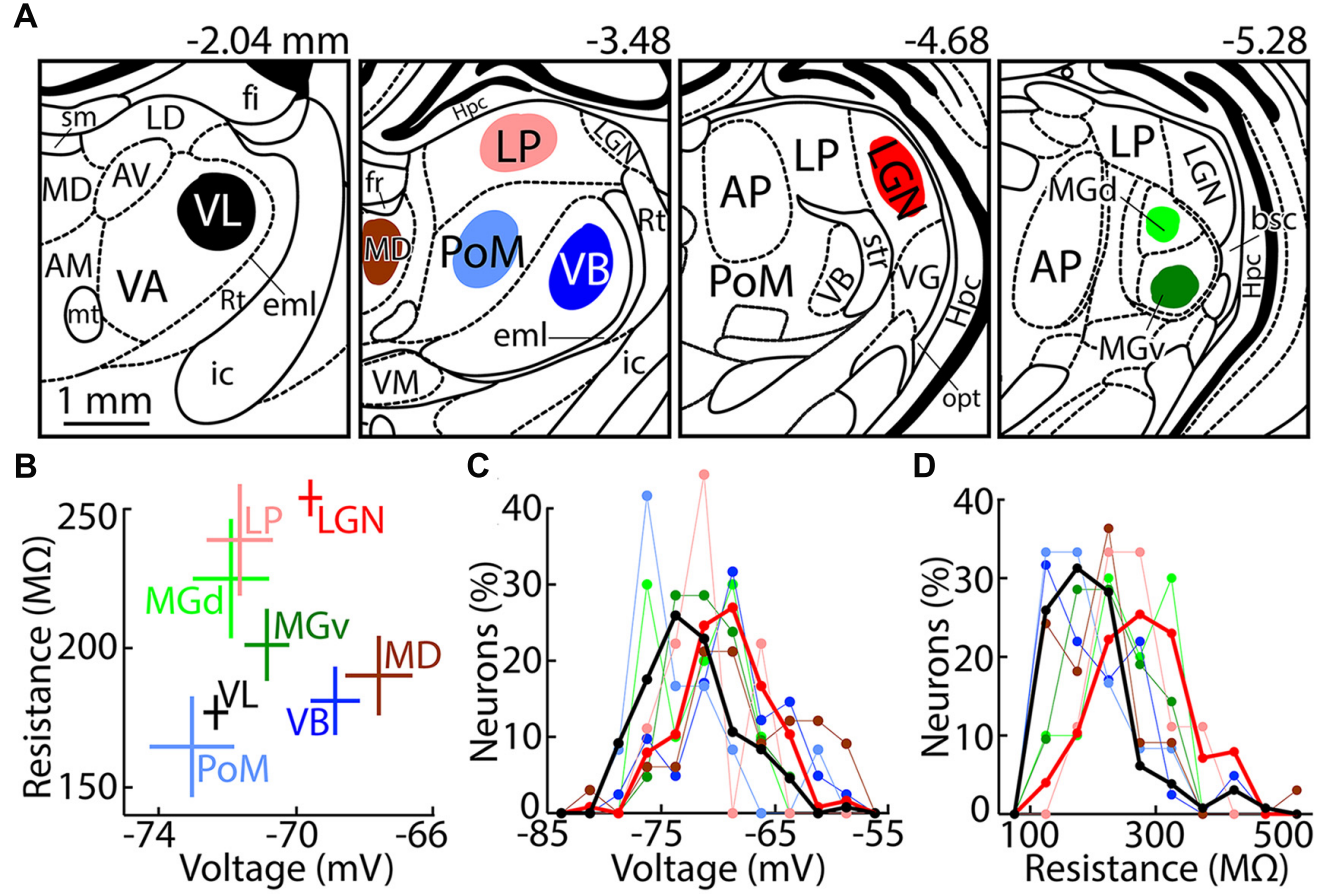
**Recording locations and passive membrane properties. (A)** Drawings of coronal sections made using the atlas of Paxinos and Watson (2007), with distance from Bregma indicated above. Neurons were recorded in the colored regions, as listed in **Table 1**. Nuclei that are labeled but were not recorded are AM (anteromedial), AP (anterior pretectal), AV (anteroventral), LD (laterodorsal), Hpc (hippocampus), Rt (reticular thalamus), VA (ventral anterior), VG (lateral geniculate nucleus, ventral division), and VM (ventromedial). Fiber tracts are bsc (brachium superior colliculus), eml (external medullary lamina), fi (fimbria of the hippocampus), fr (fasciculus retroflexus), ic (internal capsule), mt (mammillothalamic tract), opt (optic tract), sm (stria medullaris), and str (superior thalamic radiation). **(B)** Population (mean ± SEM) resting membrane potential and input resistance of thalamic nuclei. Numbers of contributing neurons correspond to the totals in **Table 2**. **(C)** Histogram of resting potentials. Bin size, 2.5 mV. Thicker lines emphasize VL (black) and LGN (red). **(D)** Same as **(C)** but showing input resistances. Bin size, 50 MΩ.

The atlas of Paxinos and Watson (2007) was used for localizing thalamic nuclei. Coronal slices of 0.25 mm were cut sequentially from rostral to caudal. Four regions of thalamus were targeted along the rostrocaudal axis (**Figure 1A**). Because neuronal membrane properties may vary with localization of neurons within a given nucleus, and because identification of atlas coordinates in living tissue can be challenging, we describe our recording locations in detail.

Ventral anterior (VA) and ventral lateral (VL) thalamus are poorly distinguished in rats using standard histological techniques (e.g., Kuramoto et al., 2009). The entire extent of VA–VL is -1.5 to -3.2 mm, with all regions caudal of -2.3 mm being labeled solely as “VL” (Paxinos and Watson, 2007). We estimate that our VL neurons were recorded over a range of -1.7 to -2.2 mm (**Figure 1A**). Caudal regions of VL were avoided because they have a long border with ventrobasal thalamus (VB). The rostral extent of VB (-2.2 mm, “ventral posterior lateral”) is lateral of VL. VB appeared darker than VL due to fibers in the external medullary lamina (eml; which blocked passage of light), but the boundary was not very distinct and thus we chose to target the region of VL just beyond the rostral extent of VB. Beyond the rostral extent of our targeted area in VL (-1.6 mm), VA–VL was smaller and appeared relatively dark, Rt became thicker (as it curved around the rostral end of thalamus), and the mammillothalamic tract (mt) was nearly invisible.

Within slices containing VL, recordings were made 2.0–2.8 mm lateral of the midline and 5.3–6.2 ventral of Bregma. The border of VL with Rt was our primary landmark (**Figure 1A**). Our VL neurons were within 1.0 mm of Rt, and within 0.6 mm in more rostral slices in which VL is smaller (**Figure 1A**). Although VL is not clearly distinguished from VA, we targeted VL by selecting neurons in the dorsolateral region of VA–VL, and we avoided the rostral pole where VA and VL are smaller and closer together (-1.6 mm caudal of Bregma). We therefore expect that at least the majority of our neurons received synaptic excitation from cerebellum, since cerebellar afferents are in dorsolateral and caudal regions of VA–VL, whereas neurons in ventromedial and rostral regions receive driving excitation form cortex and synaptic inhibition from basal ganglia (Kuramoto et al., 2009, 2011). However, the more medial of our recordings (2.0–2.2 mm) may have included some neurons that received driving excitation from cortex (Kuramoto et al., 2009, 2011). This issue is relevant to the classification of our VL neurons as “higher” vs. “lower,” as discussed below.

Our target region for VB, PoM, LP, and MD was -3.1 to -3.9 mm from Bregma. LGN served as a landmark in selecting slices, since it has an easily visualized border surrounding it (although we did not record from this rostral portion of LGN). Cutting was performed from rostral to caudal, and 1 mm of tissue was discarded caudal to our target region in VL, starting at about -2.1 mm. LGN was not visible in the next slice (it was presumably present but very small), but typically appeared in the subsequent slice at about -3.5 mm and was larger in the third and most caudal slice from this target region.

Ventrobasal neurons were recorded up to 1.0 mm from Rt, similar to the method described above for selecting neurons in slices of VL (**Figure 1A**). VB displayed a distinctive pattern created by fibers that appeared to radiate outward from near the center of the thalamus, causing it to appear darker than the more medial and dorsal PoM. Neurons in LP were recorded 0.1–0.8 mm ventral of the dorsal boundary of the thalamus. MD could be distinguished by its location on the midline and ventral of the fasciculus retroflexus (fr). Neurons were recorded at least 0.2 mm from the inferred lateral and ventral boundaries of MD. Similarly, neurons in PoM were recorded at least 0.2 mm from its inferred boundaries, the most visible of which separated it from the darker VB.

Lateral geniculate nucleus extends from -3.2 to -5.4 along the rostrocaudal axis, and slices were taken near the center where it is large in area (-4.1 to -4.8 mm). LGN was easily recognized because of its location at the dorsolateral edge of thalamus, and dark borders that separate if from adjacent thalamic nuclei. The more caudal slices from our LGN target region contained the small rostral portion of medial geniculate (MG), which is also easily recognized (see below). We recorded from dorsal LGN to avoid neurons in ventral LGN that are not thalamocortical. Most of the boundary of dorsal LGN was easily distinguished. The ventromedial boundary was more difficult to see, although the oval shape of dorsal LGN allowed its position to be inferred. Recordings were made at least 100 μm from the inferred ventromedial boundary.

Medial geniculate is between -4.8 and -6.5 mm along the rostrocaudal axis, and we targeted the central region between -5.5 and -6.0 mm. It was recognized by a distinctive pattern of dark regions surrounding it, as well as a dark band passing through it mediolaterally, dividing MGv from MGd (**Figure 1A**). Neurons were recorded near the center of each division, at least 0.05–0.10 mm from the surrounding boundaries.

### Ranking Thalamic Nuclei along Sensory-motor and High–low Dimensions

We performed correlation analyses based on sensory-motor (schemes 1–4) and high–low classifications (schemes 5–7). We considered two fundamentally different ways of classifying neurons along the sensory-motor dimension. The conventional sensory-motor classification (schemes 3 and 4) implies a symmetry in which the boundary should be near the middle of any sensory-motor pathway (a neuron may be connected to sensory input and motor output by many paths of various lengths, but we consider only the shortest path to be relevant to classifying a neuron). Regardless of the total number of synapses from sensory to motor peripheries, a neuron would therefore be sensory or motor depending on whether it is closer (separated by fewer synapses) to sensory receptor cells or motor effectors. An alternative view is that the boundary should tend to be some number of synapses (‘distance’) from the motor effector, and thus it is at least conceivable that a pathway may not include any sensory neurons. The rationale for this “distance from motor output” is based on the belief that the sensory-motor dimension is asymmetric insofar as motor output should take precedence over sensory input. For example, motor output develops prior to sensory input (Sanes et al., 2011), and it is likely that the nervous system evolved in the direction of motor to sensory (Guillery and Sherman, 2011). The key consequence of this issue for our analysis concerns the classification of somatosensory thalamus. It is naturally ‘sensory’ in the conventional classification (schemes 3 and 4), but we classified it as ‘intermediate’ based on distance from motor output (schemes 1 and 2). Schemes 1 and 2 differ only in the categorization of higher auditory and visual thalamus as ‘intermediate’ (1) vs. ‘sensory’ (2). Scheme 3 includes an ‘intermediate’ category, whereas scheme 4 has only ‘sensory’ and ‘motor’ groups.

Previous work has categorized thalamic nuclei as higher vs. lower. Sherman and colleagues have distinguished “first-order” TC neurons that receive driving excitation from subcortical afferents from “higher-order” TC neurons that receive driving excitation from layer 5 corticothalamic projections (Sherman and Guillery, 1998; Ramcharan et al., 2005; Sherman, 2007, 2012; Varela and Sherman, 2007, 2009). An apparently related distinction, based on morphology and axonal targets, has been made between C-type and M-type TC neurons (as well as less common subtypes), which predominate in lower and higher thalamic nuclei, respectively (Jones, 1998; Clascá et al., 2012).

Categorization of each of our eight nuclei as ‘higher’ or ‘lower’ was relatively straightforward, with the exception of VL. Neurons throughout most of VL are C-type and receive driving excitation from cerebellar afferents (Anderson and Turner, 1991; Kuramoto et al., 2009, 2011; Clascá et al., 2012; Nakamura et al., 2012). However, those in the rostroventromedial portion of VA–VL are M-type, receive inhibition from basal ganglia, and are driven by excitation from a source other than cerebellum, which may or may not derive from cortex (Kuramoto et al., 2011; Nakamura et al., 2012). We therefore considered three possibilities: that our sample of neurons was almost all ‘lower’ (scheme 5), ‘higher’ (scheme 6), or a nearly even mixture of higher and lower neurons so that the population average is best understood as ‘intermediate’ (scheme 7). Based on the location of our recorded neurons within VL in relation to the observations of Kuramoto et al. (2009, 2011), we believe that our neurons were predominately C-type neurons receiving excitation from cerebellum (see Anatomical Localization of Neurons). In addition, multiareal M-type neurons can be much smaller than C-type neurons (Penny et al., 1982; Clascá et al., 2012), although this is not necessarily the case in rat VA–VL (Kuramoto et al., 2009). Our selection of neurons was biased in favor of cells that appeared to have large soma, in part as a means of avoiding interneurons. These factors favor the likelihood that our population of neurons was best described by scheme 5, in which VL is ‘lower.’

Although the exact number of synapses separating TC neurons from muscle is not critical to our analysis, we note that neurons in VL are three synapses from muscle via synapses onto corticospinal neurons in layer 5b of primary motor cortex (Hooks et al., 2013; Hunnicutt et al., 2014). Our rough estimate is that some neurons in VB and LGN could be as few as four and five synapses from muscle, respectively, and it is likely that none are more than seven synapses (counting along the shortest path, which always includes corticofugal projections from layer 5).

### Data Analysis

Data were analyzed using Matlab (Natick, MA, USA). Unpaired *t*-tests were used to compare population means in analyses focused solely on VL and LGN. Comparison of population means across multiple nuclei was done with ANOVA followed by Tukey’s test for pairwise comparisons. We note that Tukey’s test is conservative in the case of unequal sample sizes. Pearson’s correlation was used for examining the relation of the responses of individual neurons, and population means, to various classification schemes.

Binomial distributions were used to estimate 95% confidence intervals in cases where we wished to compare the fraction of neurons with a LTS, or burst, across nuclei. We assumed that our only relevant knowledge was the number of recorded neurons in a nucleus and the fraction of neurons with responses (LTS or burst). The maximum entropy distribution over percentage of responsive neurons (conditional on this knowledge) is binomial with parameters ‘*n*’ (number of recorded neurons) and ‘*p*’ (fraction of responsive neurons).

Sodium spike threshold was reported for spikes evoked from near -65 mV in DCSW and aEPSG protocols. Threshold was measured as the voltage at the time the second derivative of voltage was maximal. In each cell in the DCSW protocol, the mean threshold was found for currents within 100 pA of the minimal current required to cause a spike (11 spikes in response to 11 current amplitudes). In both DCSW and aEPSG protocols, only the first spike was used in the rare case of two or more spikes in response to the same depolarizing event. In the aEPSG protocol, the first spike almost always occurred following the second of two or four aEPSG in an event, and approximately 180 spikes contributed to the mean in each cell. The population mean and SEM was found using the mean threshold from each cell.

LTS threshold was found by averaging the membrane voltage at 2 ms after current offset (to avoid the effect of the transient artifact) in the fLTS voltage trace and the voltage trace in response to the next smallest current injection (10 or 50 pA less). The fLTS itself was identified as the voltage response to the smallest current injection that had a period of positive slope (averaged over a period of 10 ms) following current offset.

‘Effective spike threshold’ was defined as the minimal membrane voltage necessary to elicit one or more spikes. If no LTS was evoked by current of any amplitude, it was equivalent to the conventionally defined threshold for the sodium-based action potential (see above). An LTS causes the effective threshold to be more hyperpolarized. If under a particular condition a LTS was present in response to at least one current, and a spike was evoked by at least one current, the effective spike threshold was the average of the super-threshold voltage trace with a spike, and the sub-threshold trace without a spike, at 2 ms following current offset (which was always at least 2 ms before onset of a spike).

LTS and TtD peak amplitudes were measured as depolari zation above baseline voltage near -80 mV. The effect of spikes was minimized by changing the voltage from 1 ms before to 4 ms after an action potential peak to its threshold voltage (calculated as described above). After this correction, the peak amplitude was estimated by taking a moving average (incremented in steps of 1.0 ms) over a period of 20 ms and finding the maximum depolarization. The accuracy of this method was verified by comparing amplitudes measured before and after application of TTX in a subset of neurons.

LTS slope was found by fitting a line to voltage in each consecutive 1.0 ms periods during the rising phase of the LTS, advanced in increments of 0.5 ms, and then selecting the maximum positive slope. In most cases the fLTS caused one or more sodium spikes, and the maximum was found near the peak of the LTS and thus in close proximity to the first sodium spike. To avoid an influence of sodium spikes, voltages above -55 mV were excluded from analysis.

H-type depolarization was measured from the initial peak of the hyperpolarization to either the period between 775 and 800 ms, or 975 and 1000 ms, following onset of the artificial conductance. In the former case, an average was found in each cell across all voltage traces in which current or aEPSG was injected at 800 ms, and this average trace was analyzed as described below. In the latter case, an average was made across all voltage traces in which small currents (300 pA or less) or one aEPSG were injected at 200 ms (only small injection amplitudes were used to reduce any potential delayed effect that the transient depolarization might have on the voltage measured near 1000 ms). Using these average voltages traces we next found the time of the initial peak. This was done by fitting a line to voltage in a 25 ms moving window that was incremented in steps of 5 ms starting at the time of hyperpolarization onset. The time of the peak was taken to be the center of the first 25 ms period in which the slope was positive. Peak times ranged across all neurons between 51 and 151 ms following hyperpolarization onset. The peak hyperpolarization was taken to be the average voltage in that 25 ms period. The depolarization attributed to activation of H-type cation channels was the difference between average voltage at 775–800 ms (or 975–1000 ms) and the voltage at the time of peak hyperpolarization.

## Results

Whole-cell recordings were made from a total of 450 TC neurons in coronal slices from eight regions of rat thalamus (**Figure 1A**). Recorded nuclei are listed in **Table 1** (together with other frequently used abbreviations), and numbers of recorded neurons in **Table 2**. We previously reported data from LGN (primary visual thalamus; Hong et al., 2014). Here, we compare our data from LGN with other thalamic nuclei. We focus in particular on VL, since it is considered to be the ‘most motor’ of thalamic nuclei. We also report results from secondary visual thalamus (LP), primary and secondary somatosensory thalamus (VB and PoM, respectively), primary and secondary auditory thalamus (MGv and MGd, respectively), and a “cognitive” or “pre-motor” nucleus (MD).

### Passive Membrane Properties

Resting membrane potentials differed across nuclei (*F* = 8.8, *p* = 10^-9^, ANOVA), and neurons in VL were significantly more hyperpolarized than those in LGN (*p* = 10^-6^, Tukey’s test; **Figures 1B,C**). Although we recorded only 9–12 neurons in each of the three higher sensory nuclei, higher nuclei as a group were more hyperpolarized than their primary counterparts, whereas sensory modality did not appear to be a significant factor with respect to resting potential (two-way ANOVA: high–low *F* = 10.8, *p* = 0.001; sensory modality *F* = 0.83, *p* = 0.44). The difference between higher and lower nuclei was particularly apparent in the case of somatosensory thalamus (*p* = 0.007, Tukey’s test). Input resistance also differed across nuclei (*F* = 12, *p* = 10^-13^). Neurons in VL and VB each had substantially lower input resistance than those in LGN (*p* = 10^-7^ and 10^-5^, Tukey’s test), with most other nuclei having intermediate values (**Figures 1B,D**).

As illustrated by membrane potential and resistance (**Figures 1C,D**), all the differences we found between nuclei were in population averages, with considerable overlap across individual cells. We presume that there is substantial variability across neurons that is not related to any sensory-motor distinction. We therefore relied on population averages across neurons within each nucleus in an attempt to ‘average out’ other sources of variability so as to isolate differences that might be related to the sensory-motor dimension.

### Three Experimental Designs

**FIGURE 2:**
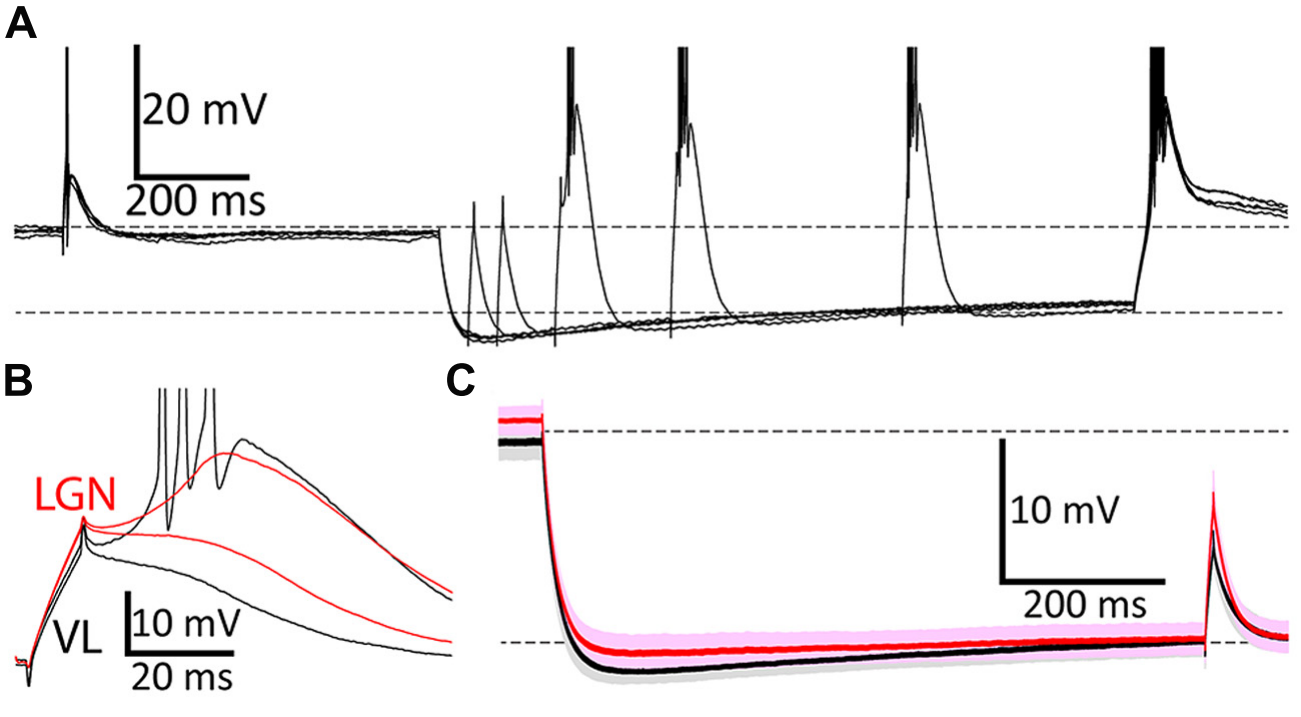
**Voltage responses during the DCSW protocol. (A)** During each of five overlaid ‘sweeps’ in a neuron in VL, membrane potential was near -65 mV (for 2.0 s) before and after hyperpolarization to near -80 mV by injection of an artificial conductance (10 nS for 1.2 s). Brief depolarizing currents (10 ms; 620 pA in this example) were injected from near -65 mV (650 ms before hyperpolarization onset) and after delays of 50, 100, 200, 400, or 800 ms near -80 mV. Dashed horizontal lines indicate -65 and -80 mV, here and in **(C)**. **(B)** Typical voltage responses from a neuron in VL (black; same cell as **A**) and another in LGN (red) after 200 ms near -80 mV to currents that were just above and below the threshold necessary to evoke a LTS. Three sodium spikes occurred in this response from VL, but their full extent is not shown. **(C)** Population average voltage in VL (black, *n* = 29) and LGN (red, *n* = 43), for the case of 200 pA injected at 800 ms after hyperpolarization. Darker and lighter shades indicate mean ± SEM and SD, respectively.

Each neuron was recorded during one of three experimental designs that varied in the extent to which they were designed to mimic natural conditions (**Table 2**). In our most natura listic design we injected artificial excitatory post-synaptic conductances (aEPSG; see below). Our “current clamp square wave” (CCSW) protocol was the simplest, with brief currents (10 ms) injected from sustained baseline potentials near -70, -80, or -90 mV that were achieved by current injection. Under natural conditions, the hyperpolarization that deinactivates TtCC in LGN, and the depolarization that activates TtCC, are caused by transient synaptic conductances (Wang et al., 2007), and these conductances decrease membrane excitability by shunting inward currents. We mimicked this by using “dynamic clamp” to inject an artificial conductance in both our “dynamic clamp square-wave” (DCSW; **Figure 2**) and aEPSG protocols. The artificial conductance hyperpolarized the membrane from approximately -65 to -80 mV, and it reduced effective resistance by about one half, similar to previous studies (Ulrich and Huguenard, 1997a; Wolfart et al., 2005; Deleuze et al., 2012).

To compare TtCC-mediated responses it is important to minimize variance of baseline voltage. Variance across neurons was modest and means did not differ across nuclei (**Figure 2C**; “-80 mV” in CCSW, mean ± SD mV, VL -79.2 ± 1.0, LGN -79.5 ± 1.1; DCSW after 775–800 ms VL -81.8 ± 1.2, VB -81.6 ± 1.3, MD -82.4 ± 1.0, MGv -82.0 ± 1.1, LGN -81.7 ± 1.1; aEPSG, VL -81.4 ± 1.2, VB -82.1 ± 1.2, LGN -80.3 ± 1.2). However, in DCSW and aEPSG experiments, it was not possible for voltage to remain constant over time near -80 mV due to the gradual depolarization known to be caused by opening of hyperpolarization-activated H-type cation channels (**Figures 2A,C**). We chose our target to be -80 mV after 800 ms. Since H-type depolarization varied across nuclei by about 1 mV, so did the initial hyperpolarization (**Figure 2C**).

### Larger H-type Depolarization in Motor Thalamus

Although it was not our intention to characterize the H-type depolarization, we found that it was twice as large in VL as LGN (**Figure 2C**) and differed significantly across the five nuclei recorded in DCSW and aEPSG protocols (mean ± SEM, VL 1.8 ± 0.1 mV, *n* = 48, VB 1.6 ± 0.1, *n* = 40, MD 1.3 ± 0.2, *n* = 12, MGv 1.2 ± 0.2, *n* = 11, LGN 0.8 ± 0.1, *n* = 94, measured from the initial peak to 775–800 ms; *F* = 18, *p* = 10^-12^, ANOVA). The depolarization was significantly greater in both VL and VB compared to LGN (*p* = 10^-8^ and 10^-8^, Tukey’s tests). This apparent difference in amplitude did not appear to be related to differences in the time course of hyperpolarization or subsequent depolarization (**Figure 2C**). The initial peak hyperpolarization occurred at the same time in VL and LGN (mean ± SEM, VL 100 ± 3 ms, LGN 98 ± 2, VB 85 ± 3, MD 122 ± 5, and MGv 102 ± 2). We found virtually identical differences in amplitude across nuclei when we measured H-type depolarization over a later range of times (from 175–200 ms to 975–1000 ms, VL 1.8 ± 0.1 mV, VB 1.7 ± 0.1, MD 1.6 ± 0.2, MGv 1.4 ± 0.2, and LGN 0.9 ± 0.06; the latest peak observed in any cell was 152 ms).

An inverse relation was evident between the average H-type depolarization and input resistance across nuclei, with lower resistance and greater H-type depolarization in motor thalamus. Both of these observations could potentially be explained by a higher density of H-type channels in motor thalamus. We therefore looked for a correlation between input resistance (measured at resting potentials) and H-type depolarization across neurons. They were indeed correlated across all thalamic neurons, as expected given the differences in population means discussed above (*r* = -0.28, *p* = 0.001, *n* = 138). However, when we segregated neurons by nucleus, we found no substantial evidence of correlation across neurons in either VL (*r* = -0.19, *p* = 0.20, *n* = 48) or LGN (*r* = -0.14, *p* = 0.40, *n* = 43) or VB (*r* = -0.03, *p* = 0.86, *n* = 40). Thus we did not find significant evidence that the observed differences in the average amplitude of H-type depolarization and input resistance across nuclei are explained by a common mechanism. We note that a stronger H-type response and lower input resistance in VL would both be expected to counteract TtCC-mediated responses (through shunting inward current, and depolarization-induced deactivation of H-type cation channels), but TtCC-mediated responses were nonetheless observed to be stronger in VL.

### Exemplary TtCC-mediated Responses in Single Neurons

**Figure 2** illustrates typical responses in single neurons during the DCSW protocol. Currents injected after 200 ms or more near -80 mV caused a “low-threshold spike” (LTS), which was blocked by nickel (0.4 mM) in both VL and LGN and is known to be mediated by activation of TtCC (e.g., Jahnsen and Llinás, 1984a,b; Hong et al., 2014). Whereas a LTS is defined by a positive voltage slope, we use the more general term “T-type depolarization” (TtD) to refer to any depolarization induced by activation of TtCC. We denote the LTS evoked by the minimal current sufficient to evoke a LTS as the “first LTS” (fLTS). In exemplary neurons from VL and LGN, selection of responses that were just above and below threshold for a LTS shows that the fLTS was larger, it was evoked from a more hyperpolarized potential, and it caused more sodium spikes in VL (**Figure 2B**).

### Greater T-type Depolarization in Motor than Visual Thalamus

**FIGURE 3:**
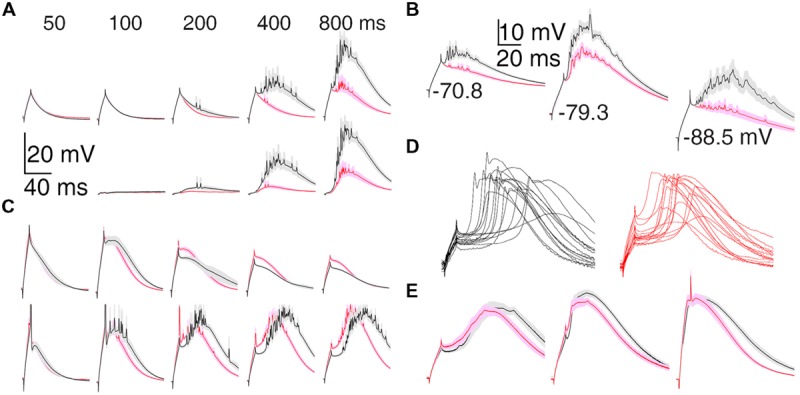
**Population average T-type depolarization is larger in motor than visual thalamus. (A)** TtD (mean ± SEM) in VL (black) and LGN (red) in the DCSW protocol. The current was selected in each cell that caused an initial depolarization (at current offset) nearest to 11.3 mV (the smallest depolarization present in all cells; range: 9.6–11.9 mV) when injected after 50 ms near -80 mV. Raw voltage responses are shown at top and isolated TtD at bottom (found by subtracting responses at 50 ms in each cell prior to averaging). Fewer cells were tested at 100 ms (**Table 2**). **(B)** Analogous to **(A)** but for the CCSW protocol. Initial depolarizations were selected to be nearly the same across all neurons but differed across the three baseline voltages. **(C)** Population average responses just below (top) and above threshold (bottom) for generation of sodium spikes. Neurons were discarded if no spikes were evoked by any current, and thus more neurons were discarded at earlier times. Similar differences between VL and LGN were apparent when analysis was restricted to neurons tested up to 1000 pA (which caused a spike in all neurons at all times). **(D)** The fLTS evoked from near -80 mV in the presence of TTX (0.5–2.0 μM) in all 30 neurons in which it was administered, including eight and seven neurons in DCSW (800 ms) and CCSW protocols, respectively, in both VL (left) and LGN (right). **(E)** For the neurons in **(D)** the population fLTS (mean ± SEM; left), the LTS in response to 100 pA more than required to evoke the fLTS (middle), and the LTS in response to our maximum tested current of 1000 pA (right; 1000 pA was tested in only 14 neurons, seven in VL and LGN, in the DCSW protocol). The fLTS was evoked by currents of 180–660 pA across all neurons. The timing of the LTS was less variable in response to larger currents, and this allows the sharp initial peak to appear in the population average with 1000 pA (right) but not smaller currents (left and middle).

Differences between motor and visual thalamus can also be seen in population average voltage responses (**Figure 3**). In response to nearly equal initial depolarizations, the average TtD was larger in VL than LGN after 200 ms and more near -80 mV (**Figure 3A**) in the DCSW protocol and from all holding potentials in the CCSW protocol (-70, -80, and -90 mV; **Figure 3B**). Subthreshold responses were smaller and super-threshold responses were larger in VL (**Figure 3C**, compare top and bottom), suggesting that TtCC activation had more all-or-none character in VL and was more graded in LGN.

A higher fraction of neurons in VL exhibited a LTS under conditions of modest TtCC deinactivation (at 50–200 ms in the DCSW protocol and -70 mV in the CCSW protocol). At 100 ms, 10 of 11 neurons had a LTS in VL but only 14 of 26 in LGN. To further quantify this difference, we estimated in each cell the elapsed time near -80 mV (DCSW), or the hyperpolarization (CCSW), that was necessary to allow an LTS to be evoked with a probability of 0.5 (by a depolarizing current of any amplitude). In VL, both the elapsed time and hyperpolarization was significantly less than in LGN (77 ± 9 vs. 110 ± 7 ms, *n* = 11 and 26, *p* = 0.02; -72.3 ± 0.7 vs. -74.5 ± 0.5 mV, *n* = 52 and 63, *p* = 0.007).

The LTS was also larger in VL than LGN when sodium channels were blocked by tetrodotoxin (TTX). TTX (0.5–2.0 μM) revealed a small and transient initial peak and subsequent hyperpolarization (2–5 mV peak to trough in 1–2 ms) in some cells (**Figure 3D**). It was larger and more prevalent in response to larger currents, at -80 vs. -70 mV, and after 800 vs. 200 ms near -80 mV, suggesting it was the result of strong TtCC activation (rather than sodium channels not blocked by TTX). It was observed on the fLTS in 10 of 15 neurons in VL at 800 ms, but in only 3 of 15 neurons in LGN, and it tended to be of larger amplitude in VL (**Figure 3D**).

The LTS tended to occur later (start and peak times) and last longer (duration and decay times) in VL (and MD) than in LGN (and VB and MGv) in the DCSW protocol, both in the presence and absence of TTX (**Figures 3C,E**). However, this was not apparent in the CCSW protocol. LTS time and duration were both sensitive to multiple factors and did not appear to have a simple relation to deinactavation of TtCC. Therefore we do not present analyses of LTS timing here. We note that the time course did not appear to vary as substantially in our aEPSG protocol (see below; Hong et al., 2014).

### Lower LTS Voltage Threshold in Motor Thalamus

**FIGURE 4:**
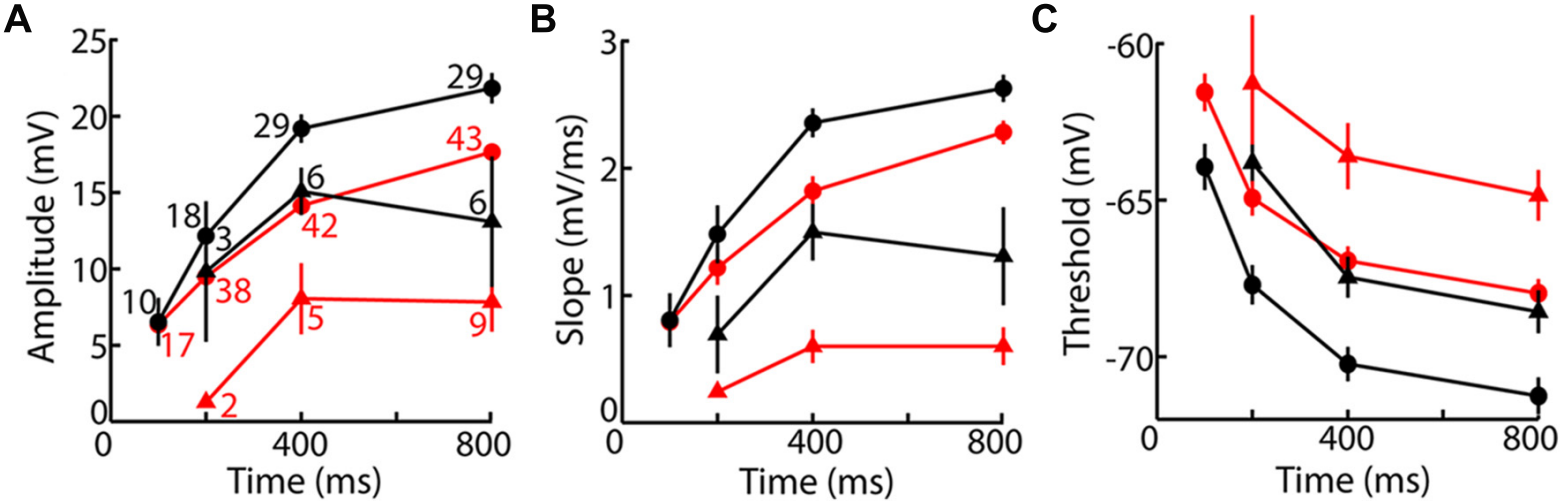
**The fLTS is more powerful in motor than visual thalamus. (A)** Population fLTS amplitude (mean ± SEM) as a function of time near -75 mV (triangles) and -80 mV (circles) in VL (black) and LGN (red). Amplitude was measured after deleting sodium spikes and performing a moving average across a period of 20 ms (see Materials and Methods). The number of cells contributing to each data point is indicated, and is the same in **(B,C)**. A LTS could not be evoked in some cells after 100 and 200 ms at -80 mV, but similar results were observed when analysis was restricted to cells that displayed a LTS at all time points. **(B)** Analogous to **(A)** but showing maximum slope of the fLTS. **(C)** Analogous to **(A)** but showing fLTS voltage threshold. Because some cells were only tested up to a maximum current of 500 pA (**Table 2**), and no sodium spike was observed in some of these cells after 200 ms or less near -80 mV (11/15 in VL, 3/12 in LGN), it is possible that a larger current would have caused a LTS with a high voltage threshold. To avoid this potential bias, we performed the same analysis only on cells tested with a maximum current of 1000 pA (which evoked a sodium spike in all cells at all times). The same result was obtained except that average LTS thresholds in both VL and LGN were more depolarized by 1–2 mV.

Population average fLTS amplitude, slope, and voltage threshold showed the expected dependence on time and voltage, and they differed between VL and LGN (**Figure 4**). Whereas the amplitude and duration of the LTS depend on a relatively complex interplay of multiple membrane properties, its voltage threshold and rising slope have a more direct dependence on the density and intrinsic properties of TtCC. Voltage thresholds were more hyperpolarized and slopes were greater in VL compared to LGN (**Figures 4B,C**), suggesting a higher density of TtCC in motor thalamus. By examination of the LTS thresholds (**Figure 4C**), we estimate that the fraction of functional (deinactivated) TtCC in LGN after 800 ms at -80 mV is similar to that observed 600 ms earlier in VL (after 200 ms at -80 mV) or 5 mV more depolarized (after 800 ms at -75 mV). TTX had no clear effect on fLTS threshold or slope (see **Figure 3E**).

### Similar Membrane Excitability with TtCC Inactivated

**FIGURE 5:**
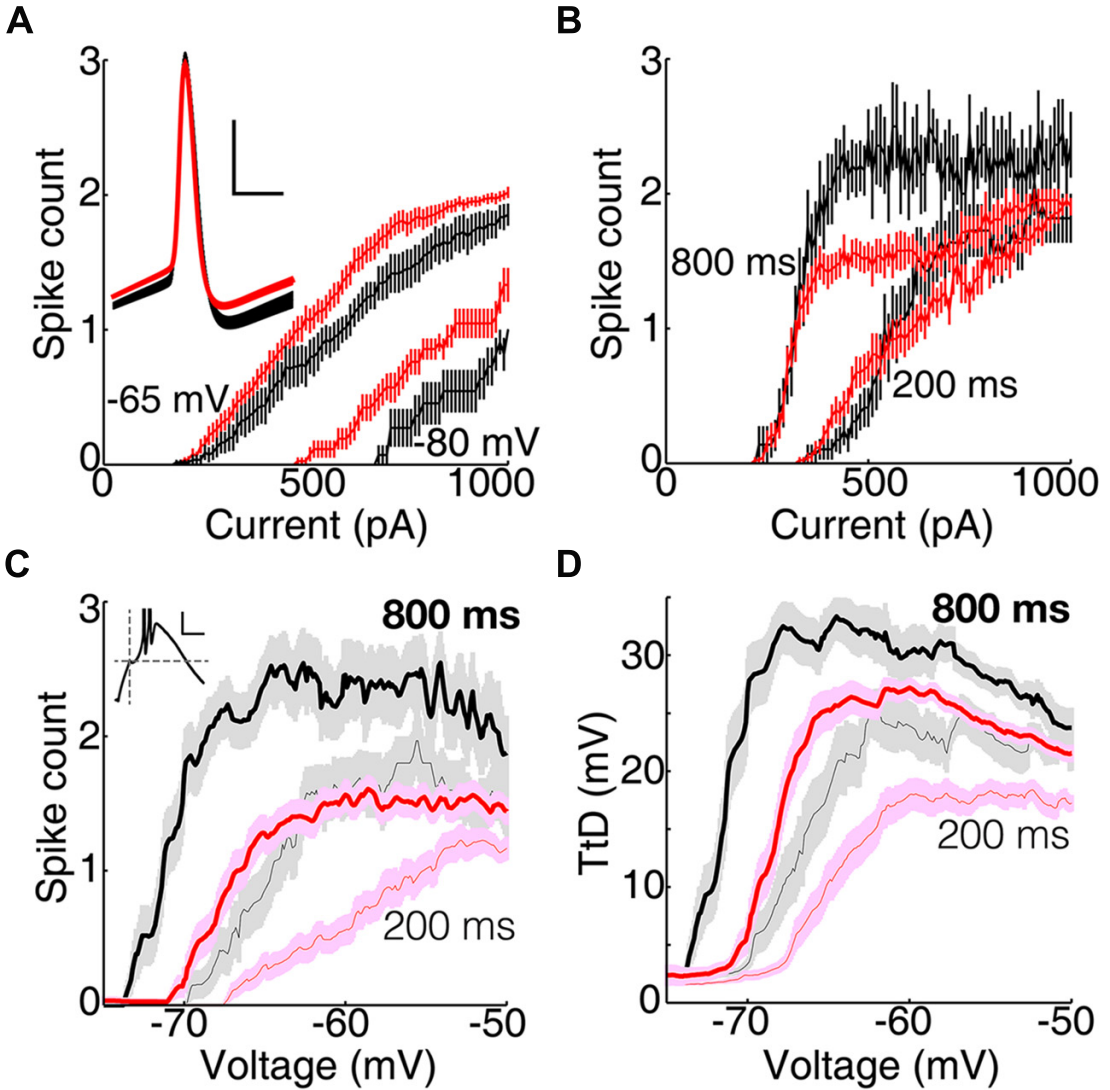
**T-type calcium channel activation causes more spikes in motor than visual thalamus. (A)** Population mean (±SEM) firing rate as a function of current injected from near -65 mV and after 50 ms near -80 mV. Only a subset of 11 of 29 and 26 of 38 neurons in VL (black) and LGN (red), respectively, were tested at -65 mV. Inset, population average action potentials (mean ± SEM) in VL and LGN selected to have peaks between 4.5 and 5.5 ms after onset of current injection from -65 mV (scale bar: 2 ms, 20 mV). **(B)** Same as **(A)** but for currents injected after 200 and 800 ms at -80 mV. **(C)** Same as **(B)** but as a function of initial depolarization. Inset, response from a single cell with dashed lines marking the time (10 ms) and amplitude of the initial depolarization (scale bar, 20 ms, 10 mV). **(D)** Same as **(C)** but showing population amplitude of the isolated TtD.

Not surprisingly, the stronger LTS in motor than visual thalamus caused a larger number of sodium spikes (see below). We attribute this primarily to TtCC because excitability was similar or lower in motor than visual thalamus under conditions of TtCC inactivation (and when TtCC were blocked by 0.4–1.0 mM nickel; not shown). For currents injected from near -65 mV and after 50 ms near -80 mV, population mean spike counts increased in an approximately linear manner in both VL and LGN, but fewer spikes were generated in VL in response to equal current (**Figure 5A**). This could most readily be explained by the lower input resistance in motor thalamus (**Figure 1**).

Action potentials had similar durations (0.7 ms at half peak amplitude) but thresholds tended to be slightly more hyperpolarized in VL than LGN (**Figure 5A**, inset; -51.9 ± 1.0 and -49.7 ± 0.7 mV, *p* = 0.09, unpaired *t*-test, for action potentials evoked by near minimal currents injected from -65 mV in the DCSW protocol; see Materials and Methods). The average after-hyperpolarization was as large or slightly larger in VL (**Figure 5A** inset). Our maximal current (1000 pA) caused an average of about two spikes in LGN, and only slightly less in VL (**Figure 5A**). Inter-spike intervals were similar in VL and LGN, even for spikes caused by a LTS (see below).

### More TtCC-evoked Spikes in Motor than Visual Thalamus

Maximal spike counts were greater in VL than LGN when TtCC were deinactivated (**Figure 5B**), opposite of the results with TtCC inactivated (**Figure 5A**). This was in spite of the lower input resistance in VL, which should reduce spike count in response to equal current. We therefore examined spike count (**Figure 5C**) and TtD amplitude (**Figure 5D**) as a function of initial voltage (at the end of the 10 ms current injection) and found that both were evoked from a more hyperpolarized membrane potential in VL than LGN, consistent with the lower LTS threshold voltage (**Figure 4C**).

The greater TtCC-evoked spike counts in VL were the result of a longer period of spiking rather than a higher frequency. The interval between first and last spikes was longer in VL than LGN (12.3 ± 0.9 vs. 5.1 ± 0.2 ms, mean ± SEM, *n* = 90 and 93 after 800 ms or more near -80 mV in DCSW and CCSW protocols, *p* = 10^-12^), whereas the interval between first and second spikes was not different, either in the case of maximal spike counts (4.8 ± 0.2 vs. 4.9 ± 0.1 ms) or in response to the smallest current that evoked at least two spikes (4.7 ± 0.14 vs. 5.0 ± 0.14 ms, *p* = 0.16).

According to theory, spike count should increase as a maximally graded function of input (current in these experi ments) in sensory neurons but should have an “all-or-none burst” character in motor neurons. The input–output (I–O) relation would be maximally graded if a minimal increment in depolarizing current were to cause a transition from 0 to 1 spike, as opposed to a “burst” of two or more spikes. Indeed, we found a significantly higher incidence of bursts (a transition from 0 to 2 spikes) in VL than LGN across each of seven conditions in DCSW and CCSW protocols (based on pairwise comparisons and binomial distributions; for example, after 800 ms or more near -80 mV, bursts were observed in 87 ± 7 vs. 42 ± 10% of 83 and 105 neurons in VL and LGN, respectively, mean ± 95% confidence interval). Similar results were obtained in our aEPSG protocol (see below). Bursts were never observed in either nucleus when TtCC were deinactivated (-65 mV and after 50 ms near -80 mV).

The LTS also had more ‘all-or-none’ character in VL insofar as it was more reliable in causing spikes. Compared to LGN, the fLTS in VL caused spikes in a higher percentage of neurons across all five times in the DCSW protocol and three holding potentials in the CCSW protocol, although the difference was statistically significant only at -70 and -80 mV in the CCSW protocol (at -70 mV, 40 ± 15 vs. 15 ± 15%, mean ± 95% confidence interval based on binomial distribution, *n* = 42 and 20, respectively; at -80 mV, 90 ± 6 vs. 62 ± 10%, *n* = 78 and 77).

### TtCC-mediated Responses across Thalamic Nuclei

**FIGURE 6:**
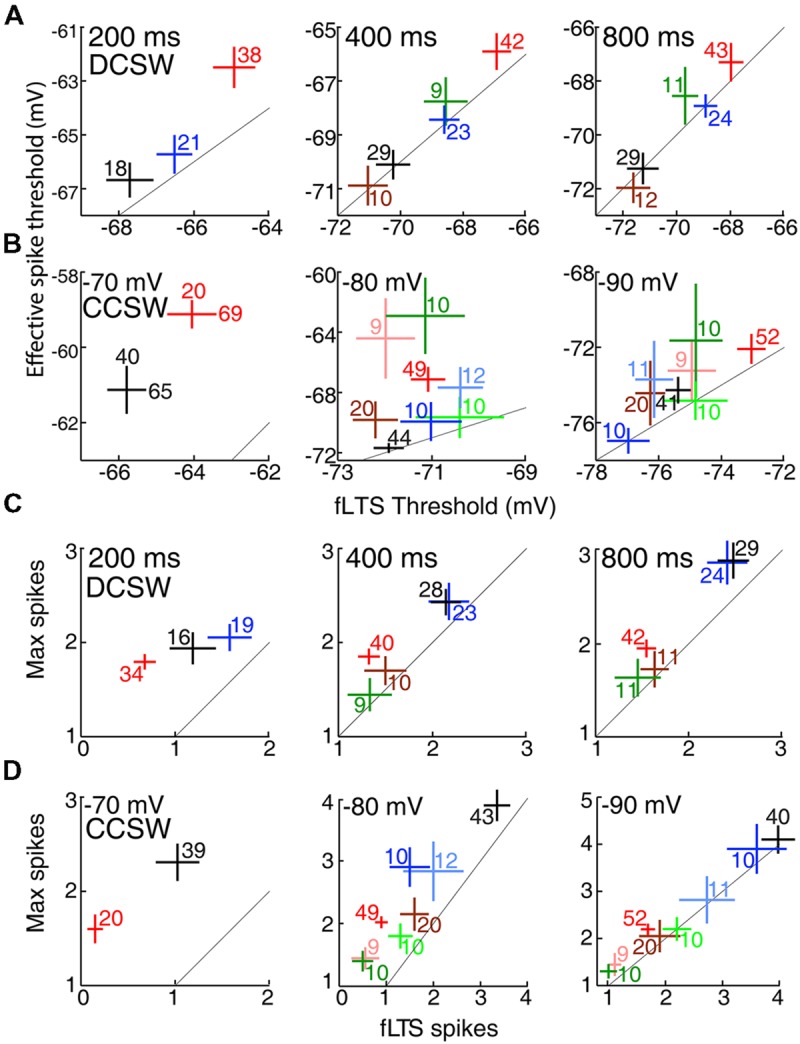
**Lower voltage thresholds and more spikes in motor than sensory thalamic nuclei. (A)** Voltage thresholds (mean ± SEM) across thalamic nuclei after 200, 400, and 800 ms (left to right) at -80 mV in the DCSW protocol. Color code as in **Figure 1**. Diagonal lines indicate identity. The numbers of cells that had a LTS and thus contributed data along the x-axis are shown. The number of cells in which spikes were evoked was within ±2 of those displaying a LTS in all nuclei under all conditions. Insufficient data was available for MGv and MD at 200 ms. **(B)** Same as **(A)** but for currents injected from -70, -80, and -90 mV (left to right) in the CCSW protocol. Sufficient data at -70 mV was available only for VL and LGN, and two inset numbers are shown because, unlike the cases of -80 and -90 mV, spikes could be evoked in the absence of a LTS in many neurons and thus we were able to measure effective spike threshold in a larger number of neurons than fLTS threshold. **(C)** Spike counts (mean ± SEM) after 200, 400, and 800 ms (left to right) in the DCSW protocol, evoked by the fLTS and in response to the current that caused the maximum spike count (usually but not always the largest current tested). Cells with no LTS were excluded from each measure of spike count. **(D)** Same as **(C)** but showing CCSW data.

Neurons in other thalamic nuclei generally had properties distributed between those of VL and LGN. **Figure 6** compares mean voltage thresholds and spike counts across all eight nuclei in six conditions. Auditory and higher visual thalamus tended to be similar to LGN, whereas somatosensory thalamus and MD had properties that were intermediate or closer to those in VL. Although primary somatosensory thalamus (VB) had similar or slightly lower voltage thresholds than its visual and auditory counterparts (**Figures 6A,B**), it had substantially higher spike counts (for fLTS spikes, *p* = 0.0008 and 0.005 vs. LGN and MGv) that were similar to motor thalamus (**Figures 6C,D**). Similarly, motor and somatosensory thalamus also had a greater number of spikes in the “rebound burst” that followed the sudden offset of hyperpolarization in the DCSW protocol (e.g., **Figure 2A**; VL 3.8 ± 0.3, VB, 3.8 ± 0.3, MD 2.6 ± 0.3, LGN 2.2 ± 0.1, MGv 1.8 ± 0.3, mean ± SEM).

TtCC-mediated responses had more all-or-none character in motor thalamus. In each of the 12 plots of **Figure 6**, the two measures are naturally correlated with one another, and the diagonal lines indicate identity. If the fLTS were to cause an all-or-none burst of spikes in all neurons of a particular nucleus, and thus larger currents were not able to evoke additional spikes, then the mean for that nucleus would lie on the identity line. The distance of population means from the identity line is a measure of the ‘gradedness’ of spike generation, which was naturally greater under conditions of modest rather than strong TtCC deinactivation (compare left and right columns). Means from VL appear closer than LGN to the identity line [except perhaps in the case of -90 mV, which is unlikely to be a physiologically relevant membrane potential given that the chloride reversal potential of TC neurons was measured to be -81 mV (Ulrich and Huguenard, 1997b)]. For example, at -80 mV in the CCSW protocol (**Figure 6D**, middle), a maximum of 1.1 ± 0.1 additional spikes were evoked by larger currents in LGN (compared to the fLTS), but only 0.5 ± 0.1 in VL.

### Mimicry of Natural Patterns of EPSPs

Although the above results are consistent with our central hypothesis, the hypothesis applies specifically to natural patterns of synaptic input *in vivo*. To better simulate natural patterns, we delivered aEPSG via dynamic clamp. We previously reported the results of these experiments in LGN, where we found that TtCC restore optimal homeostatic excitability but do not cause bursts (Hong et al., 2014). We also provided an extensive justification for the design of our aEPSG protocol based on naturally occurring patterns of synaptic input observed *in vivo* in LGN. We expect that our aEPSG protocol is not as well matched to natural conditions in VL, but in the absence of data from *in vivo* intracellular recordings in VL (see Discussion), it is not clear how it could be made more realistic.

**FIGURE 7:**
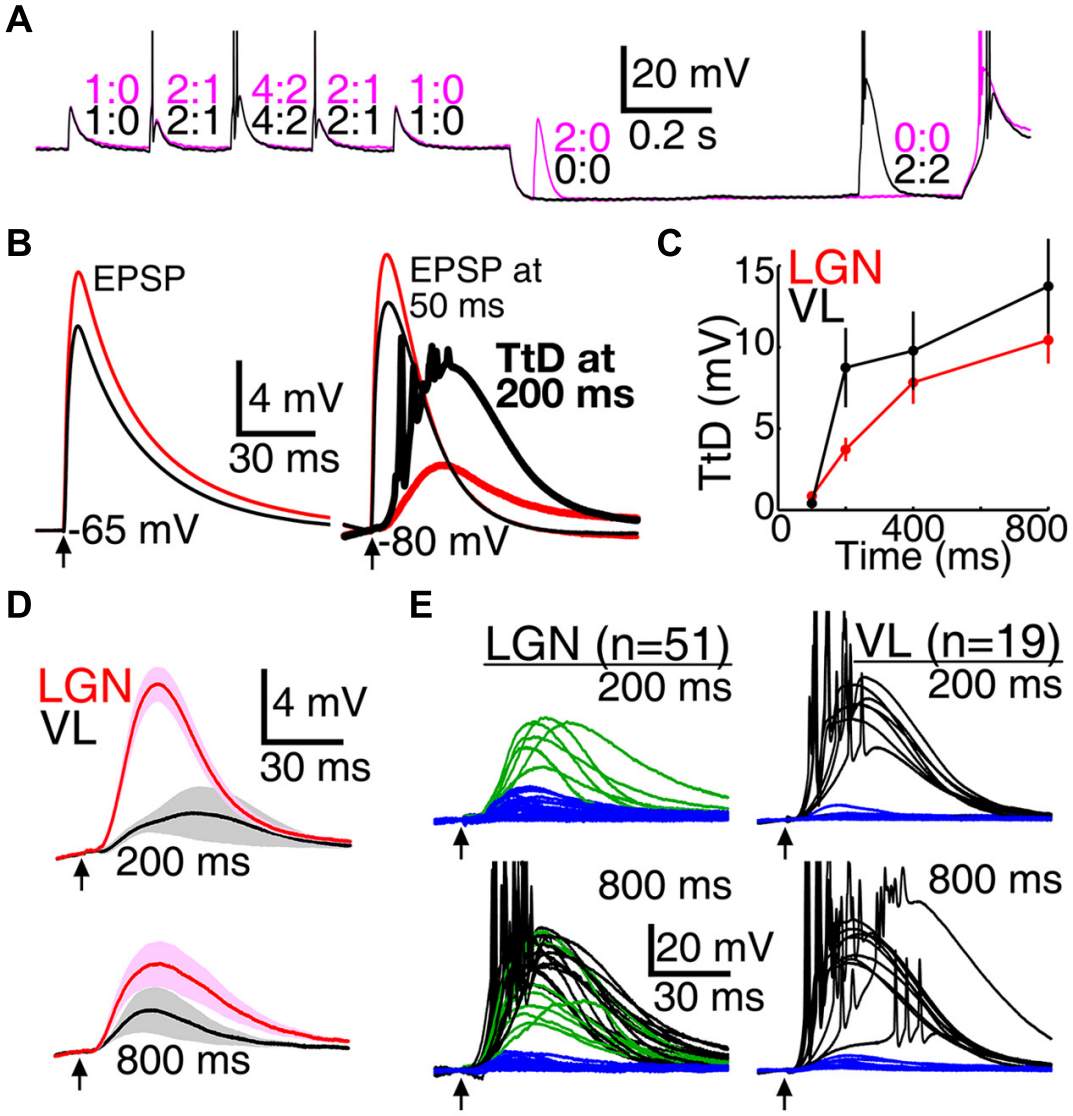
**Larger T-type responses in motor than visual thalamus during mimicry of natural synaptic input. (A)** Two voltage traces in a single neuron from VL in which an event consisting of two aEPSG occurred at 50 (magenta) or 800 ms (black). Numbers indicate aEPSG input and spike output counts for each aEPSG event. **(B)** Population mean aEPSPs (thin traces, VL black, LGN red) evoked by one aEPSG from near -65 mV (left) and after 50 ms near -80 mV (right), and the isolated TtD at 200 ms (thick traces at right, found by subtracting the voltage response at 50 ms from that at 200 ms). The TtD was larger in VL than LGN despite the EPSP being smaller, and it sometimes evoked spikes (reduced in amplitude by averaging). **(C)** Population TtD peak amplitude (mean ± SEM) in response to 1 aEPSG as a function of time near -80 mV. **(D)** Maximum isolated population TtD (mean ± SEM) that did not cause spikes at 200 (top) and 800 ms (bottom), selected in each cell without regard to aEPSG count. Cells with spikes in response to all aEPSG events were excluded; of 51 and 19 cells in LGN and VL, respectively, 51 and 12 contributed data at 200 ms, and 36 and 9 at 800 ms. Arrows indicate time of aEPSG onset. **(E)** Isolated TtD from each individual neuron in response to one aEPSG at 200 (top) and 800 ms (bottom) in LGN (left) and VL (right; averaged across four repetitions per cell). Each TtD was categorized according to whether it evoked one or more spikes (black; in response to at least one of four repetitions), was large (>15 mV) but did not evoke spikes (green), or was small (blue). All large TtD in VL caused spikes.

Mimicry of natural conditions in LGN neurons is relatively feasible given knowledge obtained from simultaneous *in vivo* recordings of visually evoked retinogeniculate excitation, feedforward inhibition, and spike output. Retinogeniculate EPSPs are large, they do not vary substantially in amplitude, and temporal summation of two EPSPs is typically required for spike generation (see *Artificial Conductances*, Materials and Methods). To mimic the natural amplitude of retinogeniculae EPSG, we adjusted the amplitude of aEPSG in each neuron so that two aEPSG of identical amplitude separated by 5 ms caused one spike from a membrane potential of -65 mV. We then delivered aEPSG events consisting of 1, 2, and 4 aEPSG (5.0 ms inter-aEPSG intervals) following a similar design to our DCSW protocol (**Figure 2**), with events delivered from -65 mV or after 50 to 800 ms near -80 mV (**Figure 7A**).

Similar to our other protocols, population average TtD were larger in VL than LGN (**Figures 7B,C**), and had more “all-or-none” character in eliciting spikes. In VL, TtD were either small and did not cause spikes (**Figure 7D**), or they were large and did cause spikes (**Figure 7E**). In contrast, large TtD were often observed in LGN that failed to cause spikes (**Figures 7D,E**; in response to one aEPSG, 7/7 and 10/10 large TtD caused spikes at 200 and 800 ms, respectively, among 19 VL neurons tested; in LGN, 0/6 and 13/21 large TtD caused spikes among 51 neurons tested).

**FIGURE 8:**
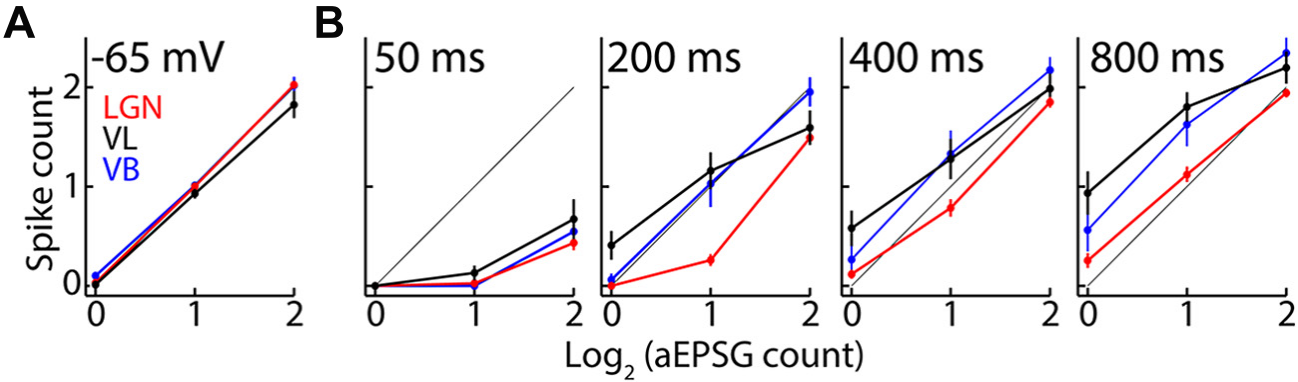
**T-type calcium channel activation alters the input-output mode in motor but not visual thalamus. (A)** Population spike counts (mean ± SEM) as a function of aEPSG count (log base 2) delivered from -65 mV for VL (black; *n* = 19), LGN (red, *n* = 51), and VB (blue, *n* = 16). The thin diagonal line (obscured by data) represents the average I–O relation of one spike per two aEPSG observed *in vivo* in LGN. The amplitude of aEPSG injected from -65 mV was adjusted in each cell to achieve this relation. **(B)** As in **(A)** but for aEPSG delivered from -80 mV after delays of 50, 200, 400, and 800 ms (left to right).

Thalamocortical neurons in LGN have an average of 1 output spike per 2 retinal input spikes (Sincich et al., 2007; Weyand, 2007), and we proposed that this is the optimal and homeostatic I–O relation in sensory neurons (Fiorillo et al., 2014). Our aEPSG protocol was designed to match this I–O relation in each cell from a baseline voltage of -65 mV (**Figure 8A**). As expected, hyperpolarization to near -80 mV initially prevented spike generation and thus flattened the I–O relation (**Figure 8B**, ‘50 ms’ at left). Deinactivation of TtCC in LGN restored the I–O relation toward its homeostatic ideal so that by 800 ms it was very similar to the I–O relation at -65 mV (compare **Figures 8B**, right, to **8A**), as previously reported (Hong et al., 2014). By contrast, TtCC deinactivation in VL caused the I–O function to shift up and to the left, becoming steeper and approaching an “all-or-none burst mode” (note that the I–O function would appear more ‘S-shaped’ if we had plotted data for zero spikes in response to zero EPSG). The I–O function in VB was intermediate but more similar to that of VL than LGN.

Bursts were more prevalent in VL than LGN. We previously reported an almost complete absence of TtCC-mediated bursts in response to retinogeniculate EPSPs as well as aEPSG in LGN (Hong et al., 2014). Here, we define a burst as an increment of two or more spikes in response to the minimal increment of one aEPSG. Only 8% of neurons in LGN (4/51) exhibited any bursts at 800 ms (in response to at least 1 of 12 aEPSG events per cell, consisting of four repetitions for each of the three aEPSG counts), whereas bursts were observed in 58% (11/19) and 38% (6/16) of neurons from VL and VB, respectively.

We previously reported similar results in LGN under additional conditions, including real retinogeniculate EPSPs in addition to artificial EPSPs, perforated-patch in addition to whole-cell patch, inter-aEPSG intervals of 2 and 10 ms in addition to 5 ms, during cholinergic receptor activation, and in the presence of reduced extracellular calcium (1.2 in addition to 2.0 mM) (although cholinergic activation and lower calcium did have significant effects) (Hong et al., 2014). However, when the aEPSG protocol was performed with only currents and not artificial conductances, TtCC often caused bursts in LGN after 800 ms at -80 mV, and the I–O function was shifted up and to the left compared to the case of -65 mV (Hong et al., 2014). We did not test these additional variables in VL or other thalamic nuclei.

### Ranking Thalamic Nuclei along the Sensory-motor Dimension

Although the data described above support our hypothesis, here we attempt a more precise test by trying to quantify the relative location of thalamic nuclei along the sensory-motor dimension and then asking how well that location explains our data. This raises several challenging issues that we summarize here and explore in greater detail in Materials and Methods.

We expect that for any particular series of neurons connected by feedforward synapses from sensory receptors to motor effectors, there should be a boundary such that all neurons on one side will be motor, and neurons on the other side sensory. However, neither our theory nor the conventional sensory-motor classification specifies where the boundary should lie. A consequence of this uncertainty is that for the purpose of data analysis we describe our limited knowledge by a multi-level ranking of nuclei, even though our theory proposes the existence of a binary sensory-motor distinction at the cellular level. An additional reason for using more than two categories is that some nuclei may consist of a mixture of sensory and motor cell types (see below).

We considered a total of seven classification schemes for thalamic nuclei (**Table 3**; see Materials and Methods for rationale). Schemes 1–4 rank thalamic nuclei along the sensory-motor dimension whereas schemes 5–7 distinguish ‘higher’ from ‘lower’ regions. Our four sensory-motor classifications are further divided into those based on the conventional sensory-motor distinction (3 and 4) and those based on “relative distance from motor output” (1 and 2). The latter schemes classify somatosensory thalamus as intermediate between sensory and motor. Our sample of neurons in somatosensory thalamus could be a mixture of neurons having sensory and motor phenotypes, favoring a classification as ‘intermediate’ (schemes 1 and 2) rather than ‘sensory’ (schemes 3 and 4; **Table 3**; see Discussion).

**Table 3:** Classification of thalamic nuclei for correlation analysis.

Scheme	Motor/high	Intermediate	Sensory/low	Mean *r*^2^	*p* < 0.05	Minimum (log)	Maximum	Mean
1	VL	VB, PoM, MD	LGN, MGv, LP, MGd	0.39	8 of 9	-16	0.07	0.02
2	VL	VB, PoM, MD, LP, MGd	LGN, MGv	0.38	8 of 9	-14	0.07	0.02
3	VL	PoM, MD, LP, MGd	LGN, MGv, VB	0.27	7 of 9	-14	0.40	0.08
4	VL, MD		LGN, MGv, VB, PoM, MGd, LP	0.22	6 of 9	-11	0.42	0.11
5	MD, PoM, MGd, LP		LGN, MGv, VB, VL	0.02	1 of 6	-2	0.84	0.45
6	VL, MD, PoM, MGd, LP		LGN, MGv, VB	0.20	6 of 9	-7	0.42	0.11
7	MD, PoM, MGd, LP	VL	LGN, MGv, VB	0.14	4 of 9	-5	0.71	0.21

**FIGURE 9:**
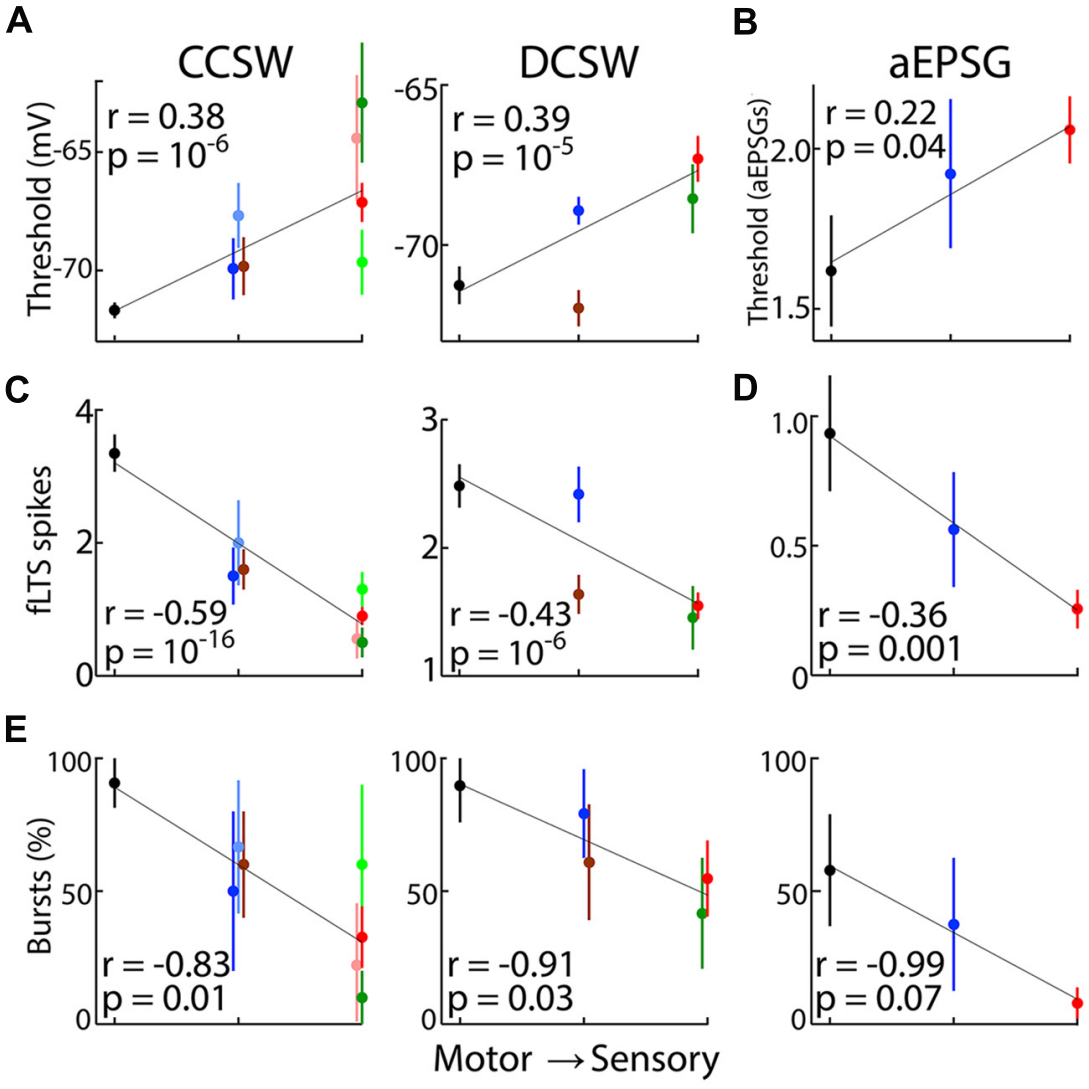
**Correlation of TtCC-mediated responses with distance from motor output.** Population mean responses after 800 ms or more near -80 mV as a function of ‘relative distance from motor output’ of thalamic nuclei as ranked according to scheme 1 (**Table 3**). Although means are shown, lines were fit and Pearson’s correlation was performed across all individual neuronal responses, except in **(E)**. Data in **(A,C)** is also shown in **Figure 6. (A)** Effective voltage thresholds (mean ± SEM) for evoking spikes in CCSW (left) and DCSW protocols (middle). Data points are slightly offset horizontally to avoid overlap. **(B)** The number of aEPSG (1, 2, or 4 in each cell) required to evoke one or more spikes after 800 ms near -80 mV (used as an alternative to voltage thresholds, which were difficult to measure in aEPSG experiments). **(C)** Spike count in response to the fLTS in CCSW (left) and DCSW (right) protocols. **(D)** Spike count in response to one aEPSG. **(E)** Percentage of neurons having a burst in CCSW (left), DCSW (middle) and aEPSG protocols (right), with ‘bursts’ defined as in Results. Unlike **(A–D)** correlations were across populations since burst incidence is not defined for single neurons. Vertical bars indicate 95% confidence intervals based on a binomial model, whereas standard error of the mean is shown in **(A–D)**.

*Correlation analysis was performed between each of the seven classification schemes and nine data sets of **Figure 9** (except six data sets for scheme 5, since only VL, LGN, and VB were recorded in aEPSG experiments and they are all classed as ‘lower’). Schemes are ‘distance from motor output’ (1–2), the conventional sensory-motor distinction (3–4), and high–low (5–7). Statistics are the mean correlation coefficient, number of *p*-values below threshold for significance, and minimum, maximum, and mean *p*-values. Mean values are not actually values of *r*^2^ and *p*, but they can be compared to one another. We present them as a crude but concise summary.*

### Correlation of TtCC-mediated Responses with the Sensory-motor Dimension

**Figure 9** shows correlations for three types of TtCC-related responses (effective spike threshold, fLTS spikes, and percent of neurons with bursts) in each of our three protocols as a function of relative distance of nuclei from motor output. Correlations across all neurons were highly statistically significant for each of the four cases in which a large number of neurons were recorded from five or more nuclei (*p* < 10^-5^; **Figures 9A,C**), and were evident but weaker for the other five cases with less data (*p* = 0.01–0.07; **Figures 9B,D,E**). Because far more neurons were recorded in VL and LGN than other nuclei in DCSW and CCSW protocols (**Table 2**), a difference between these nuclei alone could result in the appearance of a significant correlation across neurons from all recorded thalamic nuclei. We therefore repeated analyses using only the mean value for each nucleus (**Figures 9A–D**), just as we had already done for ‘burst incidence’ (**Figure 9E**). Correlations were significant (*p* < 0.05) for five of the nine data sets and nearly significant in the others (range of ‘*p*’ from 0.0007 to 0.14).

**FIGURE 10:**
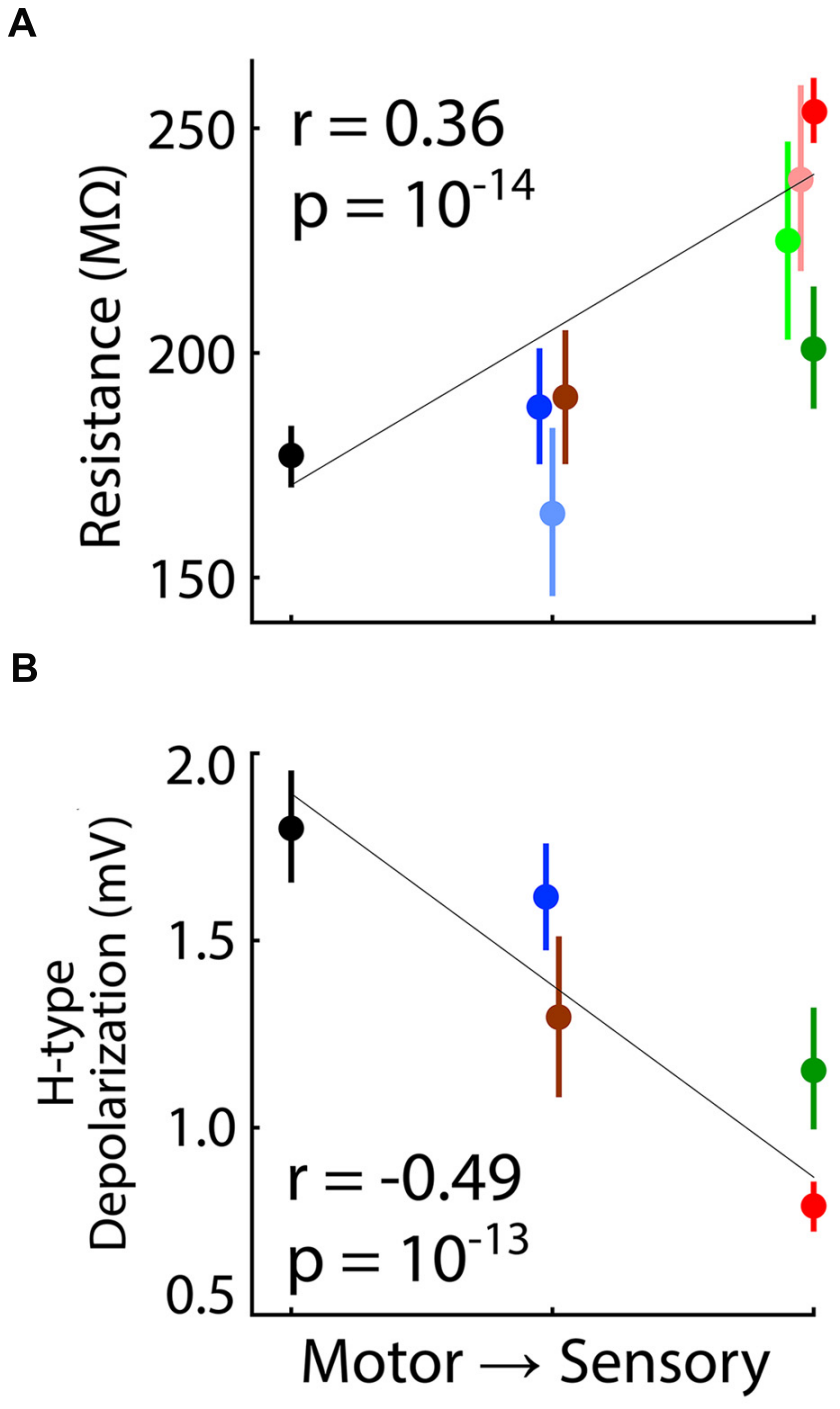
**Correlation of input resistance and H-type depolarization with distance from motor output.** Format analogous to **Figure 9. (A)** Input resistance and **(B)** H-type depolarization were correlated with distance from motor output as ranked according to scheme 1 (**Table 3**). Values of ‘*r*’ and ‘*p*’ are shown for correlation across all individual neurons, but correlations were also significant across population means.

Significant correlations were observed across all neurons and across population means of nuclei for both input resistance (**Figure 10A**; for scheme 1, *p* = 10^-14^, *n* = 383 neurons; *p* = 0.02, *n* = 8 nuclei) and H-type depolarization (**Figure 10B**; *p* = 10^-13^, *n* = 206 neurons; *p* = 0.04, *n* = 5 nuclei). Resting membrane potentials were also correlated with distance from motor output across all single neurons (*p* = 10^-5^). However, this correlation appears to have been dependent on the relatively large number of neurons recorded in VL and LGN and the difference between them (**Figures 1B,C**), since no correlation was apparent across population means (*p* = 0.90).

To compare alternative classification schemes we analyzed the nine data sets (**Figure 9**) after ordering thalamic nuclei according to each of our seven schemes. **Table 3** summarizes ‘*r*^2^’ and ‘*p*’ values for each scheme. Schemes 1–4 ranked nuclei along the sensory-motor dimension, and they were all as well or better correlated with each of our nine sets of data than any of schemes 5–7, which ranked nuclei along the high–low dimension. Based on recording locations, we suspect that most of our VL neurons probably received excitation from cerebellar rather than cortical afferents and are thus appropriately classified as ‘lower,’ corresponding to scheme 5 (see Materials and Methods). Scheme 5 showed virtually no correlation and was the weakest of the seven schemes in explaining the data. ‘Distance from motor output’ (schemes 1 and 2) was equally or more strongly correlated with each of our nine sets of data than was the ‘conventional sensory-motor’ distinction (schemes 3 and 4).

## Discussion

### Comparison of Thalamocortical Neurons across Nuclei

We have compared intrinsic membrane properties across a larger number of thalamic nuclei than previous studies (Crunelli et al., 1987; Hu, 1995; Bartlett and Smith, 1999; Leresche et al., 2004; Mooney et al., 2004; Landisman and Connors, 2007; Varela and Sherman, 2007, 2009; Broicher et al., 2008; Wei et al., 2011). We believe this to be the first comparative study of intrinsic properties to include primary motor thalamus. As predicted by theory (Fiorillo, 2013; Fiorillo et al., 2014), TtCC were more effective in causing spikes in motor than sensory thalamus.

Differences in expression of TtCC subtypes across nuclei are likely (Broicher et al., 2008), and even small differences in the properties of TtCC can have substantial effects on spike generation (Tscherter et al., 2011). A difference in TtCC density is thought to explain the greater propensity for LTS generation in higher vs. primary visual thalamus (Wei et al., 2011), and it could also account for much of our data (e.g., **Figure 4**). In particular, a moderate quantitative difference in density could explain the qualitative difference that we observed between driving membrane voltage toward vs. beyond spike threshold (**Figure 8**). However, since our primary interest here was in the relation between TtCC activation and its physiological consequences, we did not seek to characterize underlying mechanisms. Our results could be explained without any differences in TtCC across nuclei. For example, TtCC-mediated depolarization is counteracted by A-type potassium currents (Huguenard et al., 1991), which could conceivably be stronger in sensory than motor neurons.

Consistent with present and previous observations in brain slices (Wei et al., 2011), bursts of spikes have indeed been found to be more prevalent *in vivo* in higher-order visual and auditory thalamus (as well as MD) than in their first-order counterparts (He and Hu, 2002; Ramcharan et al., 2005). Our present theory and data suggest the possibility that this observed difference could be related to ‘sensory vs. motor’ rather than ‘high vs. low.’ However, regardless of that issue, it remains uncertain whether the difference in burst firing observed *in vivo* is due to differences in intrinsic properties or synaptic input, or both. Bursts in LGN result almost exclusively from bursts of retinogeniculate excitation rather than TtCC activation (Carandini et al., 2007; Sincich et al., 2007; Weyand, 2007; Casti et al., 2008; Hong et al., 2014).

The contribution of TtCC will naturally depend on the level of hyperpolarization and its temporal dynamics (see below). We found resting potentials to be more hyperpolarized in VL than LGN (∼2.5 mV; **Figures 1B,C**), although this may not have been related to the sensory-motor distinction (see Results). Although spontaneous inhibitory post-synaptic potentials were very rarely observed in our brain slices (∼2% of neurons), a tonic influence of GABA could have been present, and differences in tonic inhibition have been observed between thalamic nuclei as a result of differing expression of GABA_A_ receptor subunits (Cope et al., 2005; Bright et al., 2007). Acetylcholine and serotonin cause hyperpolarization in at least a portion of neurons in higher sensory thalamus, but only depolarization in primary sensory nuclei (Mooney et al., 2004; Varela and Sherman, 2007, 2009). Likewise, more hyperpolarized resting potentials were found in explants of higher than lower auditory thalamus (Senatorov and Hu, 1997; Mooney et al., 2004), although studies in conventional brain slices generally found no difference in resting potentials between higher and lower sensory regions of thalamus (Bartlett and Smith, 1999; Landisman and Connors, 2007; Varela and Sherman, 2007, 2009).

Neurons in motor thalamus had significantly higher input conductance and stronger H-type depolarization than those in sensory thalamus (**Figure 10**). Despite a very high level of statistical significance, we interpret our observations of H-type depolarization with caution since experiments were not specifically designed to characterize it. Previous studies found that H-type depolarization was stronger in lower (MGv) than higher-order (MGd) auditory thalamus (Hu, 1995; Bartlett and Smith, 1999), but found no difference in input conductance between primary and higher-order sensory nuclei (Bartlett and Smith, 1999; Landisman and Connors, 2007; Varela and Sherman, 2007, 2009).

Neurons in primary somatosensory thalamus (VB) had properties that were intermediate on average between those of motor thalamus (VL) and those of primary auditory and visual thalamus (MGv and LGN), including strength of TtCC responses as well as input resistance and H-type depolarization. Neurons in many early somatosensory regions are more closely connected to motor regions in comparison to neurons in visual and auditory regions. Somatosensory thalamus has been shown to project to superficial layers of primary motor cortex (Hooks et al., 2013; Hunnicutt et al., 2014). There could be heterogeneity of neurons within primary somatosensory thalamus analogous to that demonstrated in primary somatosensory cortex (Yamashita et al., 2013), with some TC neurons projecting to superficial layers of motor cortex (Hooks et al., 2013) and others projecting to somatosensory cortex.

Our sample of neurons in somatosensory thalamus could have been a mixture of neurons having sensory and motor phenotypes. Two types of neurons have been distinguished in rat primary somatosensory thalamus based on voltage responses to whisker deflection (Brecht and Sakmann, 2002). One type (those excited by stimulation of multiple whiskers) was distinguished from the other by lower input resistance and stronger TtCC-mediated amplification of sensory-evoked EPSPs. These properties are similar to those we observed in motor thalamus, and could correspond to our proposed ‘motor phenotype.’

### Theory of a Sensory-motor Dichotomy

The present study extends a general theoretical framework that seeks to understand biophysical properties of neurons in relation to information and prediction (Fiorillo, 2008, 2010, 2012, 2013; Fiorillo et al., 2014; Hong et al., 2014). This theory correctly predicted that TtCC would serve a homeostatic function in maintaining the optimal “single spike mode” in LGN under natural conditions (Hong et al., 2014), in contrast to the longstanding belief that TtCC cause bursts throughout thalamus (e.g., Jahnsen and Llinás, 1984a). The theory also correctly predicted the present evidence that TtCC are more powerful in motor thalamus, where they do appear to make a causal contribution to bursts.

We propose that “active motor control” is distinguished from “passive sensory perception” by the causal contribution of TtCC and other VGC to spikes in motor but not sensory neurons. In addition to being either excitatory or inhibitory, we proposed that synapses and ion channel subtypes can be divided into two classes based on whether they have been designed (through associative learning rules or some other selection process) to have either a causal role (class 1), or non-causal and homeostatic role (class 2), in generating or suppressing spikes (Fiorillo et al., 2014). Activation of a ‘causal’ (class 1) excitatory or inhibitory ion channel (or synapse) should reliably be followed by spikes or no spikes, respectively. The classic example would be an excitatory synapse that is selected by a Hebbian ‘spike timing dependent’ learning rule to cause spikes. Hebbian rules selectively strengthen those excitatory synapses that are synchronously active and thereby sum together to cause spikes. We have proposed that Hebbian rules also regulate TtCC and some other VGC subtypes in motor but not sensory neurons (Fiorillo, 2013; Fiorillo et al., 2014). A subtype of TtCC under Hebbian control would become strong if its current sums with current from coactive excitatory synapses to cause spikes. If a developing neuron expresses a variety of TtCC or other VGC subtypes that vary in their voltage- and time-dependence, a Hebbian rule would selectively strengthen those subtypes that are most effective in amplifying EPSPs and thereby causing spikes. A neuron could thereby learn to recognize and respond to specific temporal patterns of sensory-related synaptic input.

We proposed that other ion channels and synapses have been selected to have a homeostatic (non-causal) role in spike generation, by driving EPSP peak voltage toward spike threshold so that spikes may or may not occur depending on the precise amplitude of the EPSG (Fiorillo et al., 2014). An ion channel subtype would develop this homeostatic property if its density is regulated by anti-Hebbian learning (Fiorillo, 2008; Fiorillo et al., 2014), as has been previously demonstrated for inhibitory synapses (Barlow and Foldiak, 1989; Hosoya et al., 2005). Since an anti-Hebbian rule implements negative feedback, it would weaken a TtCC subtype when its activation is followed by a spike, and would strengthen it when its activation is followed by absence of a spike. This should result in TtCC being fine-tuned to amplify EPSPs toward but not beyond spike threshold.

We provided evidence that TtCC in sensory neurons serve this homeostatic role (Hong et al., 2014). TtCC did not “cause” spikes in LGN insofar as substantial TtCC activation was not followed by spikes in many instances (**Figure 7E**), and the average number of spikes was no more than would have been caused by the same synaptic excitation from depolarized potentials in the absence of TtCC activation (**Figure 8**; Hong et al., 2014). In contrast, substantial TtCC activation in motor thalamus was almost always followed by spikes (**Figures 7E** and **8**).

### Limitations of the Present Evidence

There are multiple limitations to the ability of the present data to fully test the hypothesis. These include variability across neurons that is not related to the sensory-motor dimension, our limited knowledge of sensory-motor pathways through the thalamus, and the challenges of understanding and mimicking natural patterns of synaptic input. Recordings of membrane voltage in behaving animals could help to overcome these challenges, particularly in simpler and better understood nervous systems, such as invertebrates.

The present data is not sufficient to test the specific hypothesis that the sensory-motor distinction is a dichotomy rather than a continuum. We believe it is a dichotomy because a particular subtype of VGC (or synapse) in a single cell should be selected to have either a causal or non-causal role in spike generation (by driving EPSP peak voltage above or below spike threshold in the former case, and toward spike threshold in the latter case; see above). If the only difference between two populations of neurons were ‘sensory vs. motor,’ our model would predict bimodal distributions of membrane properties. That we did not observe bimodal distributions (e.g., **Figures 1C,D**) can be readily explained by other sources of variability in intrinsic properties. For example, neurons within a single thalamic nucleus differ in their receptive fields (e.g., Brecht and Sakmann, 2002; Vigeland et al., 2013), and presumably they therefore receive different patterns of synaptic input. Our general theory seeks to explain all the intrinsic properties of neurons as the result of adaptation to distinct patterns of synaptic input, but in practice we cannot use our theory to explain the properties of specific neurons unless we have knowledge of the patterns that they experience. We have knowledge of patterns that are likely to be similar across TC neurons (e.g., large unitary EPSPs from “drivers”; Sherman, 2007), but we have almost no knowledge of the differences across TC neurons.

We do have relatively good knowledge of synaptic patterns in LGN. The duration of hyperpolarization determines the extent to which TtCC are deinactivated, and it appears to typically be less than 0.5 s (Wang et al., 2007). After such brief hyperpolarization, we found that amplification of EPSPs by TtCC in LGN promotes homeostasis but is too weak to fully restore the optimal I–O relation (**Figure 8**; Hong et al., 2014). Virtually no recordings of membrane potential have been made in motor thalamus *in vivo* (but see Timofeev et al., 2001). If motor TC neurons experience more prolonged periods of hyperpolarization than those in LGN, this would be expected to further facilitate the causal role of TtCC in spike generation in motor thalamus, beyond the difference observed here. If instead hyperpolarized periods are substantially more transient in motor than visual thalamus, it is conceivable that TtCC may serve a homeostatic function in motor thalamus that is analogous to their role in visual thalamus. This would contradict our favored hypothesis of a sensory-motor distinction as it applies to TtCC in thalamus (though it would be consistent with the proposed need for homeostatic VGC in all neurons).

Although less informative than intracellular recordings, extracellular recordings can nonetheless help us to make inferences about the natural dynamics of membrane voltage. Average rates of excitatory retinogeniculate input and spike output in LGN are about 20 and 10 Hz, respectively (Sincich et al., 2007; Weyand, 2007; Wang et al., 2010). In motor thalamus, average firing rates were near 15 Hz (Anderson and Turner, 1991; Nakamura et al., 2012) and powerful excitatory and inhibitory afferents from deep cerebellar nuclei and basal ganglia, respectively, fire near 50 Hz or more (e.g., Hikosaka and Wurtz, 1983; Rowland and Jaeger, 2005; Blenkinsop and Lang, 2011). Average rates at least twice that high have been observed during simultaneous recordings of motor TC neurons and their inhibitory synaptic input from basal ganglia (pallidum/area X) in juvenile zebra finches (Goldberg and Fee, 2012). That same inhibitory synapse is large and powerful and can cause strong hyperpolarization (E_Cl_ = -90 mV; Luo and Perkel, 1999; Person and Perkel, 2005). Under *in vivo* conditions in which synaptic excitation from cortex presumably occurred at high rates, inhibition at this synapse caused suppression of firing that was too brief (∼5 ms) to be associated with deinactivation of TtCC (Goldberg and Fee, 2012). However, the same synapse could cause more sustained hyperpolarization in other sensory-motor contexts or later stages of development.

### Evidence of Sensory-motor Differences Outside Thalamus

The available evidence from non-thalamic neurons supports the generality of the present hypothesis. Motor cortex is more prone to generate rhythmic oscillations than somatosensory cortex (Castro-Alamancos and Tawara-Hirata, 2007), which is likely due at least in part to differences in intrinsic membrane properties between those regions (Castro-Alamancos and Tawara-Hirata, 2007; Miller et al., 2008; Castro-Alamancos, 2013). However, most of the studies relevant to our hypothesis have not directly compared sensory and motor neurons. Certain subtypes of VGC generate patterns of spikes in motor networks known as “central pattern generators,” and this can occur even in the absence of synaptic input (Marder and Bucher, 2007). In “bistable” neurons presumed to be ‘motor,’ the summed effect of excitation from VGC (such as L-type calcium channels) and synapses is required for spike generation, examples being spinal motoneurons (Carlin et al., 2000) and striatal medium spiny neurons (Wilson and Kawaguchi, 1996; Vergara et al., 2003). Whereas calcium channels and other VGC in these motor neurons promote temporal patterns, VGC have been shown to counteract temporal patterns in neurons of sensory regions such as LGN (Hong et al., 2014) and primary visual cortex (Wang et al., 2003).

Extracellular recordings of spike times also support the theory, according to which neurons of motor systems should have more temporally patterned spike output. It is well known that firing in LGN and sensory cortex appears relatively “random” (or “Poisson-like”; e.g., Softky and Koch, 1993; Dan et al., 1996). Comparison of 15 cortical regions in behaving monkeys found firing in sensory regions to be less patterned than motor regions (Shinomoto et al., 2009). Our theory and the present data offers a mechanistic explanation for this observation at the level of spike generation in single neurons.

## Conflict of Interest Statement

The proposed distinction between sensory and motor neurons was first introduced in a US patent (Fiorillo, 2013). The patented idea concerned Hebbian and anti-Hebbian associative learning rules for regulation of voltage-gated ion channels. These learning rules are briefly summarized in the Discussion. They are important to the theory, but they were not the subject of the present experiments.
